# Genetic exchange shapes ultra-small Patescibacteria metabolic capacities in the terrestrial subsurface

**DOI:** 10.1128/msystems.00046-25

**Published:** 2025-08-15

**Authors:** Emilie Gios, Olivia E. Mosley, Nobuto Takeuchi, Kim M. Handley

**Affiliations:** 1 School of Biological Sciences, The University of Auckland 1415, Auckland, New Zealand; University of Illinois Chicago, Chicago, Illinois, USA

**Keywords:** horizontal gene transfer, Patescibacteria, terrestrial subsurface, metagenomics

## Abstract

Bacterial genomes are highly dynamic entities, mostly due to horizontal gene transfer (HGT). HGT is thought to be the main driver of genetic variation and adaptation to the local environment in bacteria. However, little is known about the modalities of HGT within natural microbial communities, especially the implications of genetic exchange for streamlined microorganisms such as Patescibacteria (Candidate Phyla Radiation). We searched for evidence of genetic exchange in 125 Patescibacteria genomes recovered from aquifer environments and detected the presence of hundreds of genomic islands, individually transferred genes, and prophages combined, with up to 13% of genome length attributed to HGT. Results show that most individual gene transfer events occurred between Patescibacteria, although putative donors included phylogenetically diverse groundwater microorganisms. For example, results indicate exchange of a *lysR* transcriptional regulator gene between Omnitrophota and Patescibacteria taxa with highly similar relative abundance patterns across 16 groundwater samples. Overall, results indicate a wide variety of metabolic functions were introduced into Patescibacteria genomes by HGT, including transcription, translation, and DNA replication, recombination and repair. This study illustrates the evolutionarily dynamic nature of Patescibacteria genomes despite the constraints of streamlining and that HGT in these organisms is also mediated via viral infection.

**IMPORTANCE:**

Genomic fluidity and diversity in bacteria are mainly governed by horizontal gene transfer (HGT), leading to a variety of genome structures and physiological diversity. The predominantly uncultivated Patescibacteria comprise highly diverse bacteria that consistently exhibit small cell and genome sizes. Despite strong pressures to reduce genetic content, we predict that these ultra-small bacteria use HGT to the same extent as other bacteria and that HGT may help facilitate recovery and maintenance of critical metabolic functions, niche exploitation, and putative symbiont-host interactions. Here, we determine the contribution of gene exchange to the evolution and diversification of Patescibacteria, despite the constraints of streamlining. We provide evidence of gene gains in Patescibacteria genomes recovered from aquifer environments and describe the large extent to which ultra-small bacterial genomes are subjected to HGT. Results suggest distinct metabolic functions acquired by Patescibacteria compared to general groundwater communities, suggesting specific evolutionary pressures on gene transfer dynamics occurring in ultra-small prokaryotes.

## INTRODUCTION

The horizontal transfer of genetic material (or horizontal gene transfer; HGT), involving acquisition of exogenous DNA, is thought to be a key factor in the evolution of prokaryotes, especially in bacteria (1). HGT accounts for a significant fraction of gene diversity and genomic fluidity in these organisms, leading to a variety of genome structures and physiological diversity (2). This evolutionary process is important for niche adaptation and is often driven by mobile genetic elements (MGEs) such as genomic islands (GIs), transposons, and phages. Many routes exist for gene transfer: uptake of environmental DNA molecules (transformation), exchange between two cellular organisms via a physical bond (conjugation), or delivery through phage infection (transduction).

Recent metagenomic studies of subsurface ecosystems reveal a large group of unusual bacteria referred to as the Patescibacteria phylum (equating to the Candidate Phyla Radiation; CPR), which has been suggested to comprise up to 26% of all currently known bacterial diversity (3, 4). The group constitutes taxa consistently harboring small cell and genome sizes (5). Implications of reduced size include limited metabolic functions, such as the lack of known amino acid and nucleotide biosynthesis pathways (6). These observations led to the prediction that most ultra-small Patescibacteria have a symbiotic lifestyle (5), and some were confirmed to be episymbionts based on cultivation and microscopy studies (7). Close physical associations with other organisms due to metabolic dependencies, along with the long residence time of groundwater (8), and the importance of the biofilms in aquifers (9), could favor genetic exchange in subsurface ultra-small microbial communities.

Despite proximity to other groundwater bacteria, extensive gene loss in Patescibacteria evolution could result in selective pressures against the acquisition and retention of exogenous DNA that are stronger than in non-streamlined genomes. Another potential barrier to HGT lies in the fact that genome reduction in obligate intracellular symbionts has often been associated with loss of functions involved in DNA recombination and repair (10), which could limit integration of foreign DNA. Previous studies have, however, shown not only that a significant fraction of Patescibacteria organisms still possess genes required for homologous recombination (6), but that they are naturally competent for uptake of eDNA molecules, mediated by DNA intake pumps (via comEC and pili complexes) (6, 11, 12).

Investigating the mobility of adaptive genes via HGT could provide important information about the evolutionary strategy of these ultra-small organisms, including aspects governing putative host-symbiont interactions. Implications of HGT include altered interaction modalities with host populations, as described in *Vibrio fischeri*. Adding a single gene to the *V. fischeri* genome is sufficient to alter its host range in squids (13). In addition, acquisition of MGEs has been shown to constitute evidence of symbiotic relationships between HGT recipient and donor, such as extensive gene transfer from *Wolbachia* endosymbionts to their insect hosts during genome reduction (14), and putative acquisition of host-derived ankyrin repeat proteins by bacterial sponge symbionts to inhibit phagocytosis (15). How potentially adaptive genes aid niche adaptation in ultra-small prokaryotes and fine-tune metabolic interactions with their predicted “basibiont” prokaryotic host cells remains largely unknown.

To understand the role of HGT in the evolution of groundwater communities, and particularly streamlined and prolific Patescibacteria, we analyzed 396 unique metagenome-assembled genomes (MAGs), including 125 from Patescibacteria. The MAGs were derived from alluvial aquifers, where Patescibacteria were dominant members of the communities (16). We investigated genomic evidence for individual horizontal gene transfer events (defined here as HT genes) in the MAGs, the frequency of these events, and compared these to acquisitions of larger MGEs such as GIs and prophages. Results provide evidence for many HGT events among Patescibacteria and exchanges with other prokaryotes in groundwater.

## RESULTS AND DISCUSSION

### Prevalence of HGT events in Patescibacteria genomes

We first investigated the potential horizontal transfer of individual genes between aquifer microorganisms using the community-level HGT detection tool MetaCHIP (17). MetaCHIP determines candidate transfers via nucleotide sequence similarity and then predicts gene flow direction and validates candidate transfers via reconciliation of gene and species trees using Ranger-DTL (18). We also further validated a subset of exchanged genes by additional phylogenetic analysis. The MAG data set used was reconstructed from eight wells across two alluvial aquifers in the same geographic region and comprised only MAGs estimated to be ≥70% complete and <5% contaminated (average completeness 87.9% ± 8.4 standard deviation [SD] and contamination 1.2% ± 1.4 SD; Table S1), and that were unique following dereplication at 99% average nucleotide identity (ANI). Overall, MAGs in this non-redundant and quality-filtered data set (*n* = 396) accounted for 1.8% to 15.0% of the sampled groundwater communities (7.2% on average ± 4.4 SD, Table S2), and represented the major taxonomic groups present according to 16S rRNA gene amplicon sequence variant analysis (e.g., the 21 most abundant phyla accounting for 94%–98% of the community, Table S3). Individually, these MAGs represented a wide spectrum of the groundwater community in terms of relative abundance (7.8% to 0.003% based on mapped reads per MAG), with most present at less than 1% relative abundance (391/396 MAGs) (Fig. S1). This tendency toward low relative abundance is explained by the high community diversity typical of groundwater microbial communities (e.g., reference [19]), as also indicated by Shannon indices of groundwater samples in this study (average 6.3 ± 0.5 SD based on amplicon sequence variants) (see Gios et al. [16]). Despite the high diversity, the same prokaryotic populations were found across multiple samples. Most populations represented by MAGs (*n* = 297/396) were present in at least two samples based on a minimum genome coverage of ≥70%, 16 were present in at least half of the samples, and one Pseudomonadota (MAG nzgw537) was present in all 16 samples (average 2.8 samples per MAG ± 2.1 SD; Fig. S1). In addition, a rarefaction curve of the combined MAG data set indicated sampling neared saturation (Fig. S1). The MAG data set, while not exhaustive, therefore includes representatives of major and common taxa present in the sampled communities. As MetaCHIP identified HT genes via comparisons among the genomes provided, only individual gene transfer events that occurred among the set of 396 MAGs analyzed were inferred.

**Fig 1:**
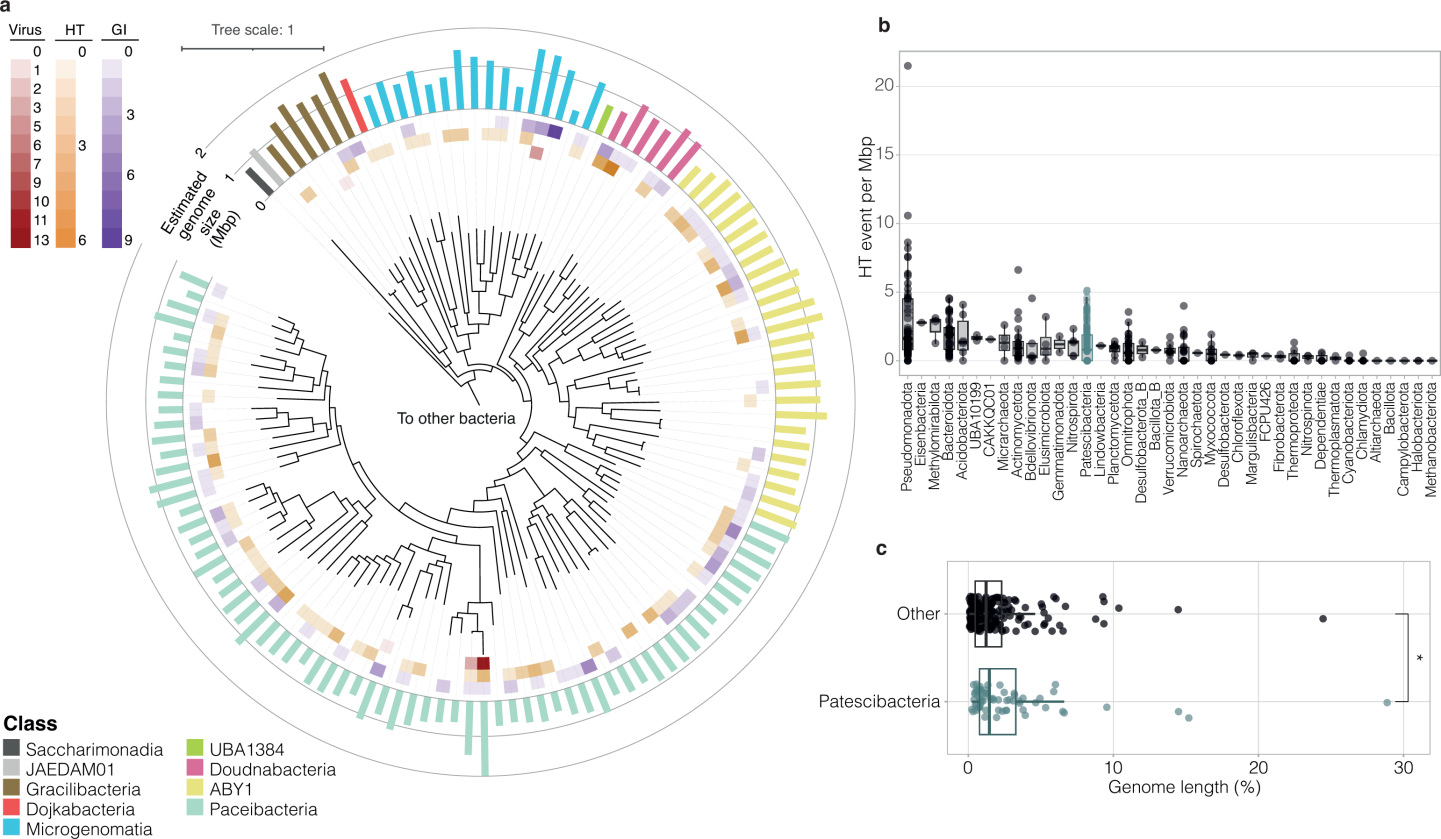
Prevalence of HGT in Patescibacteria and the wider groundwater community. (**a**) Maximum likelihood phylogenetic tree of 125 Patescibacteria MAGs recovered in this study. Tree is based on 120 concatenated bacterial marker gene alignments from GTDB-Tk. Of these marker genes, 77 ± 11.5 SD genes were used for Patescibacteria, and 101.3 ± 11.7 SD were used for non-ultra-small bacteria. Rings from the center: number of prophages; number of HT events; number of GIs; estimated genome size (Mbp). The scale bar indicates the number of substitutions per site. (**b**) Number of recipient HT events between all groundwater MAGs per phylum normalized by genome size. Patescibacteria phylum is indicated in teal. The center line of each boxplot represents the median; the top and bottom lines are the first and third quartiles, respectively; and the whiskers show 1.5 × the interquartile range. (**c**) Proportion of each genome occupied by acquired GIs and prophage sequences, comparing Patescibacteria (teal) and the rest of the community (black). Significant differences were assessed for each group using Wilcoxon Signed Rank (*: *P* < 0.05).

A total of 1,487 transfers were identified among these groundwater MAGs (273 recipient MAGs, Table S4), including 1,407 unique HT genes, where transfers may have occurred directly between the gene-exchange (donor-recipient) pairs identified, or indirectly, via an unrecovered taxon. Sequence divergences between the HT genes of gene-exchange pairs were between 3.9% and 34.5% or on average 23.7% (± 4.0 SD), suggesting most of the observed transfers were not recent. Taxonomic breakdown of detected HT events revealed that over half of the Patescibacteria genomes experienced HT with another genome in the data set, regardless of their taxonomy or estimated genome size (Fig. 1a and b).

**Fig 2:**
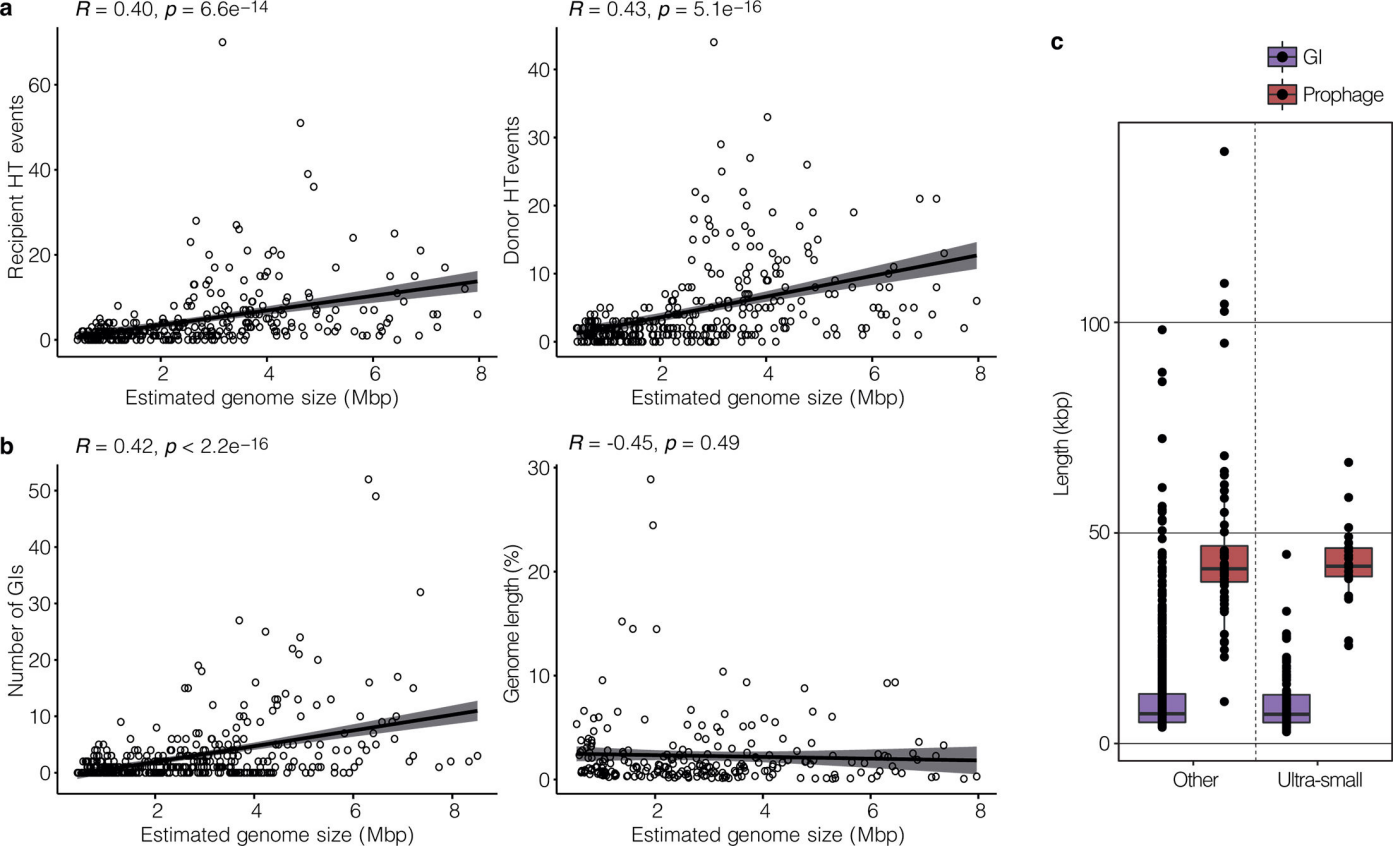
Correlations between the estimated genome size of groundwater-derived MAGs and HGT, and HGT length distributions. (**a**) Estimated genome size compared with recipient HT events (left) and donor HT events (right). (**b**) Estimated genome size compared with the number of GIs detected (left), and the proportion of genome length occupied by GI and prophage sequences (right). (**c**) Length distribution of GIs and prophages in ultra-small MAGs (i.e., Patescibacteria, Dependentiae, and Nanoarchaeota) and other groundwater microbial MAGs. The center line of each boxplot represents the median; the top and bottom lines are the first and third quartiles, respectively; and the whiskers show 1.5 × the interquartile range. No significant differences were detected in GI and prophage sequence length between ultra-small microorganisms and other groundwater microorganisms (Wilcoxon Signed Rank, *P* >0.05).

Gene flow predictions indicated 124 (9% of) genes were horizontally acquired by 68 Patescibacteria organisms, representing 1.0 HT genes received per genome (± 1.2 SD) and 1.1 HT genes per Mbp (± 1.3 SD) (Fig. 1b). In comparison, results indicated that genomes of other members of the community contained on average 3.5 ± 4.8 SD horizontally transferred genes per genome, but a comparable 1.4 per Mb ± 2.1 SD. Predictions of whether a gene was received or donated by MetaCHIP are shown to have over 80% accuracy (17), and are as such a good overall indicator of gene flow direction within a community, although the directionality of individual transfer requires further validation (examples are given below of exchange between Patescibacteria and other phyla of DNA topoisomerase, transcriptional regulator, ABC transporter, and lysophospholipase genes). The comparison of HT events per Mbp indicates that the rate of exchange of individual genes is mostly independent of genome size (i.e., larger genomes do not receive proportionally more genes). This is supported by significant positive correlations between HT events per genome and estimated genome size (both donor and recipient HT events, Fig. 2), and is consistent with previous observations of HT acquired genes in prokaryotic genomes (20), and of transfers occurring between distantly related organisms (i.e., cross-order transfers, >85% of the total HT events detected here) (21). To confirm MAG contamination did not influence findings, a comparison between HT genes received and MAG contamination estimates was undertaken, which indicated no relationship (Fig. S2).

**Fig 3:**
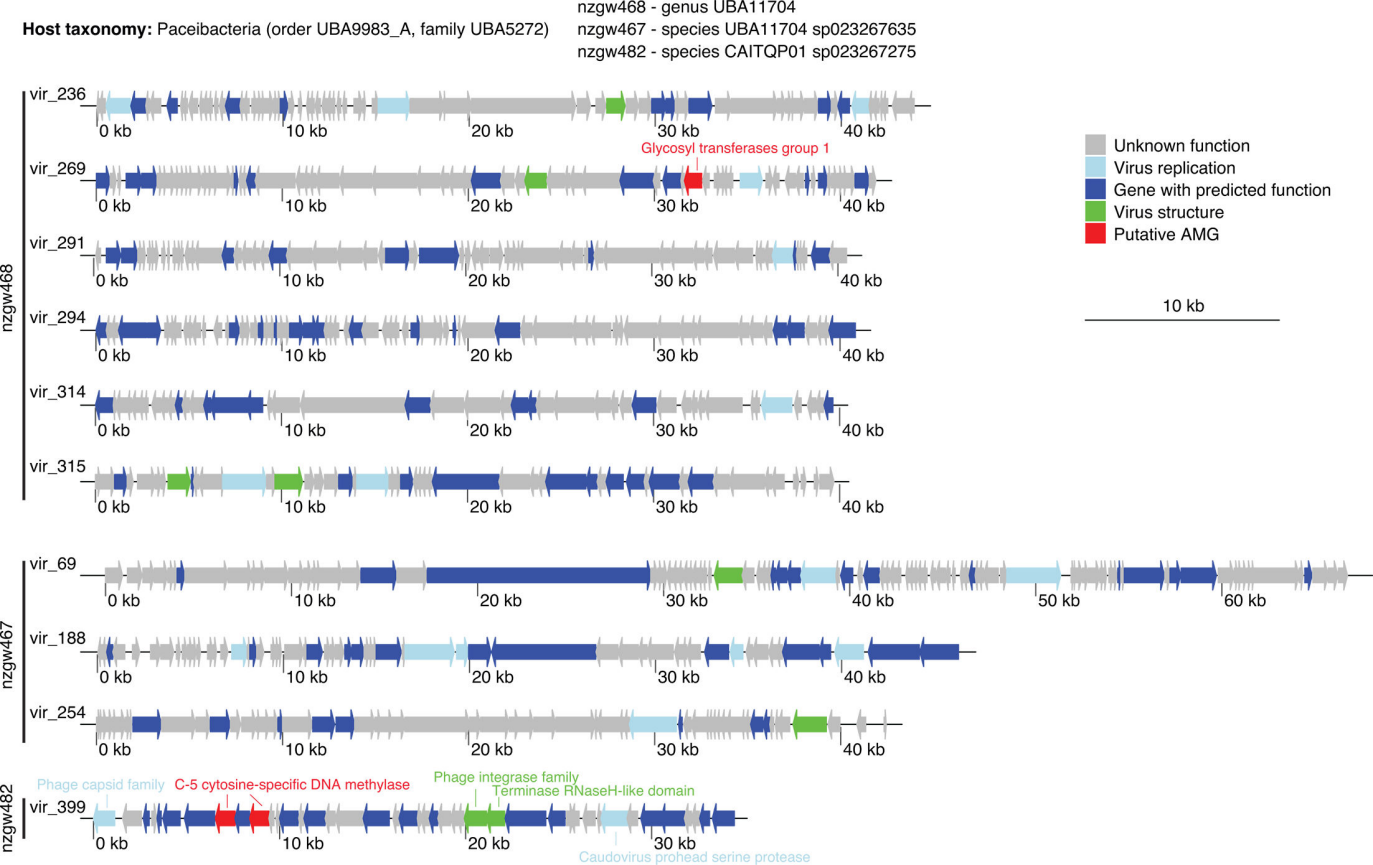
Gene plots of prophage found in Patescibacteria MAGs. The linear viral genome maps indicate the location of genes related to viral replication and structure, and of putative AMGs (auxiliary metabolic genes). Functional gene annotations were generated using DRAM-v.

When searching for large MGEs, we found 120 GIs in 63 Patescibacteria MAGs that were inferred by IslandViewer4 (1,046 across all MAGs, 228/396 MAGs). These GIs ranged from 2.7 to 45 kbp long, with 40 GIs over 10 kb long (average length 17.4 kb ± 0.7 SD) (Table S4). In addition, 10 putative prophage genomes were found to be concentrated in three Patescibacteria MAGs (all Paceibacteria family UBA5272; Fig. 3), and another four viral genomes were co-binned in Halobacteriota, Pseudomonadota, and Methylomirabiolota MAGs (6 MAGs overall). This is consistent with our previous analysis of viral distributions (lysogenic or lytic) in these groundwater samples, which showed Patescibacteria, along with Pseudomonadota and Methylomirabiolota, were the most abundant taxa groups overall, and had the highest numbers of distinct viruses (viral operational taxonomic units) based on *in silico* linkages (22). All three Patescibacteria MAGs with putative prophages were also predicted to harbor GIs, with each MAG harboring five to seven predicted prophages and GIs in total (MAG nzgw467 had three prophages and one GI, MAG nzgw468 had six prophages and one GI, and MAG nzgw482 had one prophage and four GIs). The Paceibacteria prophages were similar in length (average 43,740 bp ± 8,661 SD), and two encoded putative auxiliary metabolic genes for DNA methylation (C-5 cytosine-specific DNA methylase) and carbohydrate metabolism (glycosyl transferases group 1) (Fig. 3; Table S5), indicating gene acquisition by the phage from past hosts (23). Only prophages from Paceibacteria are reported here. However, prophages have also been reported from other members of the Patescibacteria (Absconditabacteria, Gracilibacteria, and Saccharibacteria), including those employing the host’s alternate coding (24). Therefore, multiple ultra-small lineages appear to be infected by temperate phage. Nonetheless, the high frequency of prophages detected in ultra-small bacterial genomes in this study is surprising, as prophage frequency has been shown to increase with genome size (25). Accordingly, our analysis shows that, as also observed for HT genes, the frequency of acquisition of longer complex DNA sequences, constituting GIs, increased with the genome size of groundwater-derived MAGs (Fig. 2b).

When considering the groundwater community overall, the proportion of genome length occupied by GIs and prophages, combined, remained constant regardless of recipient genome size (Fig. 2b), and despite GI and prophage sequences being similar in length between ultra-small and other microorganisms (Fig. 2c; Table S6). This is consistent with observations by Ochman et al. (1) that the proportion of horizontally transferred genes is independent of the amount of protein-coding sequences in complete prokaryotic genomes. However, when comparing the two community fractions, results here show the proportion of genome length occupied by putative GIs and prophages was slightly, but significantly, higher in Patescibacteria than the rest of the communities—on average, 2.4% ± 2.4 SD versus 1.5% ± 1.7 SD, respectively (Fig. 1c).

The extent of gene transfer events detected here (84% of MAGs contained at least one HT gene or GI) is broadly comparable to those detected in a variety of other phylogenetically diverse microbial communities, for example, 89.9% of groundwater MAGs containing detectable HGT events, compared to 78.8% in cheese-associated communities, and 89.5% in prokaryotic isolate genomes (1, 26). However, the extent of genetic exchange observed in the groundwater microbial community is likely underestimated. For example, additional HT events are likely to have occurred within populations sharing very high genomic similariy (>99% ANI). Very recent HT events were not captured (non-capture of recent HT events could be due to a methodological limitation of metagenomics with poor assembly of highly similar stretches of sequence; [17]), and ancient transfer events will escape detection due to robust integration within the recipient genome via modification of GC content and tetranucleotide frequencies (27). Moreover, while MetaCHIP has proven efficient in predicting individual gene transfer donor/recipient relationships within microbial communities, donor taxa might not have been captured in the MAG data set, due to sequencing depth (i.e., rare taxa), which is a limitation for highly diverse aquifer ecosystems (16), or temporal shifts in community structure or composition. In addition, HT genes may not be detected due to their presence in unassembled regions of MAGs.

### HGT origins in Patescibacteria taxa

**Fig 4:**
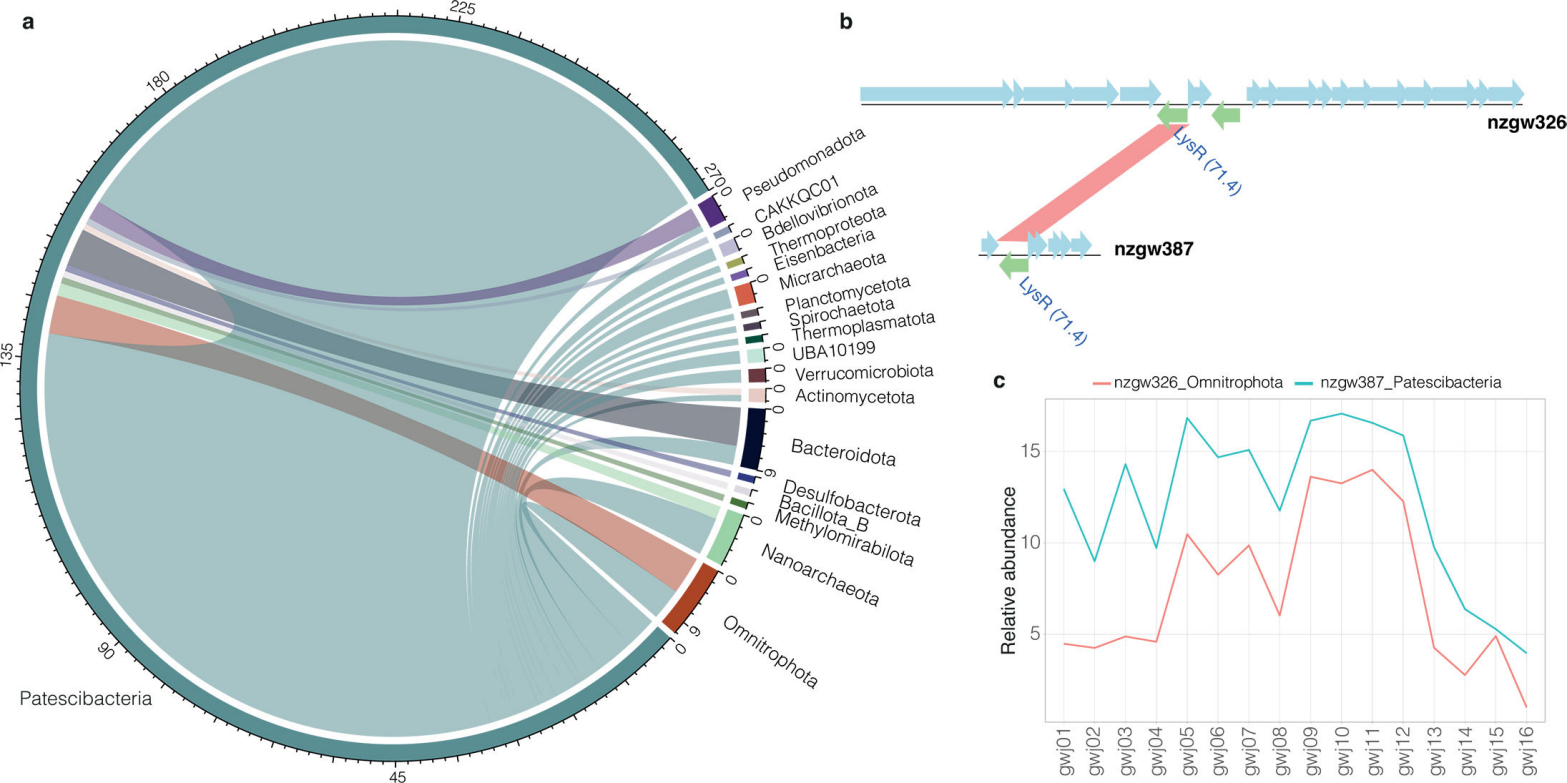
Origins of HT genes in Patescibacteria taxa. (**a**) Gene flow among phyla donating to Patescibacteria taxa. Scale represents the number of HT events, and the inner bands show the donor/recipient relationship (colored according to the donor organism). (**b**) Example of HT gene transferred from Omnitrophota nzgw326 (upper plot) to Patescibacteria nzgw387 (lower plot). Genes encoded on the forward strand are displayed in blue, and genes coded on the reverse strand are displayed in green. (**c**) Genome relative abundance profiles of these two organisms across 16 groundwater samples.

Most HT events detected are predicted to have occurred between members of the Patescibacteria (Fig. 4a), with 104 of the 124 HT genes predicted to be received by Patescibacteria originating from counterparts. This observation is consistent with previous research demonstrating that HGT is most prevalent among closely related organisms (26) and that intra-species HGT occurs at far greater frequency (28). HGT was likely also facilitated by the spatial proximity of microorganisms (29), in particular by microbial cohorts in groundwater communities (30). This includes cohorts formed by ultra-small prokaryotes (bacteria and archaea), where individuals spatially co-occur as described previously in subsurface and soil ecosystems (16, 31–33).

Although a smaller number of MAGs were recovered from Omnitrophota and Bacteroidota, organisms in these phyla are predicted to have shared genes more frequently with Patescibacteria (3.5 times and 3 times more, respectively) than, for example, Pseudomonadota, which was the most populous phylum (Table 1; Tables S3 and S7). For example, MetaCHIP results and an additional phylogenetic analysis illustrate that a member of the Bacteroidota transferred a DNA topoisomerase gene (*gyrB*) to a Patescibacteria (Paceibacteria MAG nzgw495) (e.g., Fig. S3). Frequent sharing may indicate close relationships between Omnitrophota and Bacteroidota and ultra-small prokaryotes, such as via a host-symbiont relationship or close proximity in a shared niche. Both bacterial groups have been identified previously as putative HGT donors to Patescibacteria in seafloor environments (34) and were found to consistently co-occur with ultra-small bacteria in aquifers (35).

**TABLE 1:** Frequency of HT events to Patescibacteria at phylum level^*a*^

Donor phylum	Donor HGT events	Reconstructed donor MAGs	Ratio donor HGT events/number of MAGs
Patescibacteria	109	125	0.87
Omnitrophota	5	28	0.18
Bacteroidota	6	33	0.18
Nanoarchaeota	2	23	0.09
Pseudomonadota	3	51	0.06
Actinomycetota	1	24	0.04
Methylomirabilota	1	3	0.33
Bacillota_B	1	1	1.00
Desulfobacterota	1	1	1.00
CAKKQC01	1	1	1.00

^*a*^ Note the ratio of donor HGT events to the number of MAGs is biased in donor phyla with small MAG sample sizes (Methylomirabilota, Bacillota_B, Desulfobacterota, CAKKQC01).

**Fig 5:**
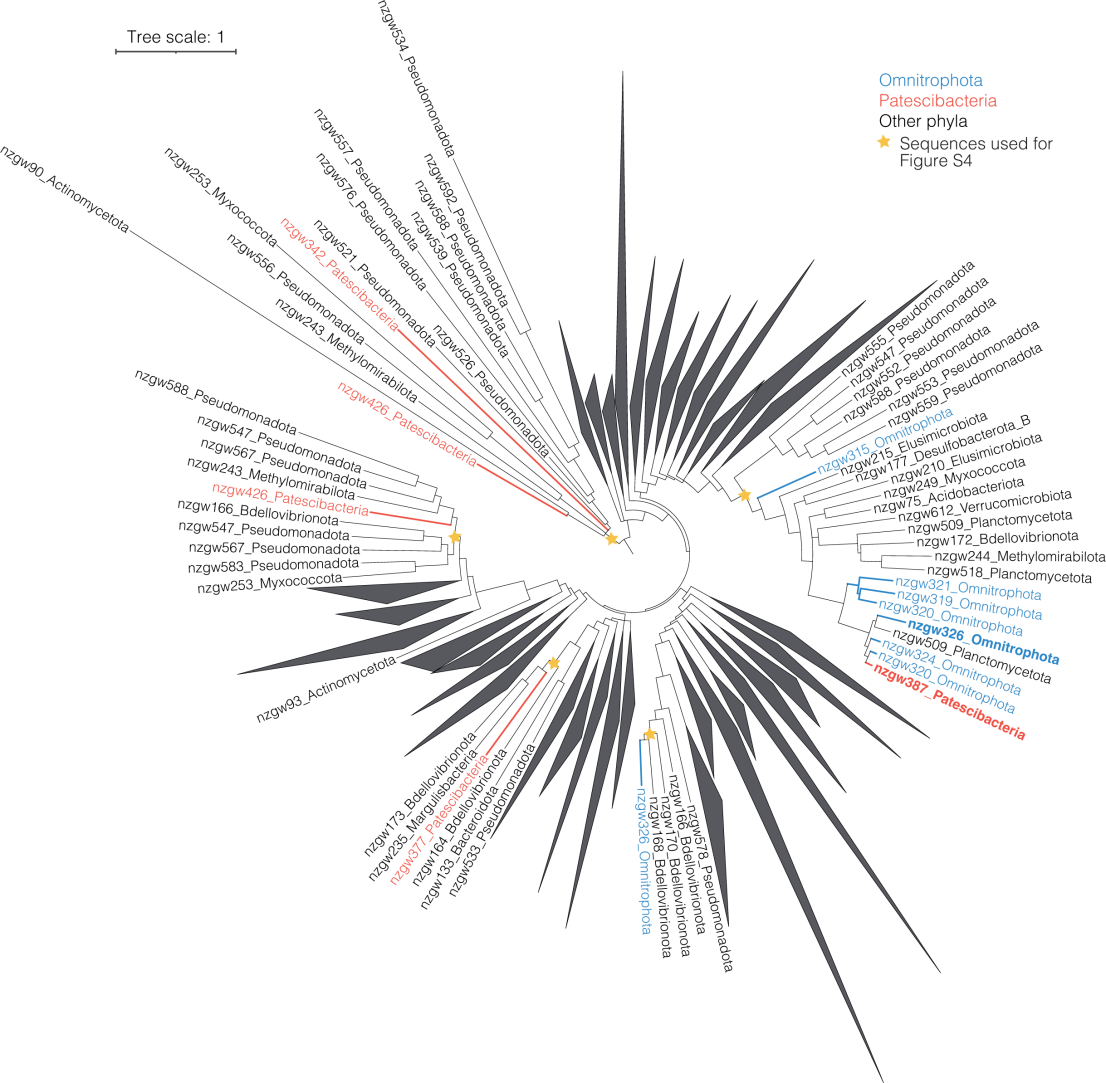
Phylogeny of LysR protein sequences. The tree uses all LysR protein sequences in this study longer than 50 amino acids long and shows the close phylogenetic relatedness between the LysR proteins encoded by Omnitrophota nzgw326, other Omnitrophota, and Patescibacteria nzgw387. Clades one node below sequences of interest were collapsed for better visualization. Sequences contained in branches marked with a star were used for generating a more robust phylogenetic tree—including all 56 LysR sequences from this study (Fig. S4).

To assess the potential for close physical associations between HGT pairs, we compared the relative abundances of each member in gene-exchange pairs involving Patescibacteria. Abundance correlations have been used previously to illustrate putative host-symbiont pairs from subsurface samples (36), although identifying such physical associations may be complicated by various factors. For example, members of the same patescibacterial species are shown to have taxonomically distinct hosts (i.e., different genera) (37). Moreover, bacteria sharing similar niches may not maintain stable abundances relative to one another. Nonetheless, results here showed significant positive correlations for 29 out of 131 pairs across aquifer sampling sites (Pearson’s correlation coefficient >0.5, *P* <0.05, Table S8). This included correlations with four Bacteroidota and Omnitrophota as putative donors (most other significant correlations were between intra-patescibacterial pairs: 24/29). As an example, results based on MetaCHIP suggest that Patescibacteria nzgw387 received a copy of the *lysR* gene from Omnitrophota nzgw326 (Fig. 4b). LysR is a highly conserved and widespread prokaryotic transcriptional regulator involved in numerous cellular functions (38). Patescibacteria nzgw387 also co-occurred spatially with its putative donor, Omnitrophota nzgw326, in groundwater (Fig. 4c), although further research is needed to determine whether this co-occurrence represents a host-symbiont relationship or a simple shared niche preference. Further phylogenetic analysis of these and other LysR predicted protein sequences via tree construction confirmed the phylogenetic placement of the Patescibacteria nzgw371 LysR next to the LysR from Omnitrophota nzgw326 and showed close relatedness to LysR encoded by several other Omnitrophota (Fig. 5; Fig. S4). Hence, it is likely that Patescibacteria nzgw371 received *lysR* from Omnitrophota. The tree also indicates a lack of phylogenetic conservation of LysR in general and extensive gene exchange between members of other phyla (e.g., Pseudomonadota) and Patescibacteria.

A BLASTP analysis of GI genes against the gene content of whole groundwater communities showed that most of the genes located on GIs could not be matched to any organisms within the communities (91.9% of GI genes; ≥30% sequence identity and ≥50% query coverage, Table S9). Nonetheless, we found that 7% of GI genes were related to those in Patescibacteria other than the one carrying the GI. The content of one 4,250 bp long GI in Patescibacteria nzgw468 (74% genome completeness, 0.0%–4.7% estimated contamination) was also highly similar to genes in the archaeon Halobacteriota nzgw24 (99% MAG completeness, 1.3%–1.9% estimated contamination), indicating a potential and relatively recent transfer event between the two distantly related organisms. None of the 11 genes located on the GI could be functionally characterized (as for a large proportion of genes in Patescibacteria genomes), but were between 85% and 100% identical in amino acid composition to the genes of nzgw24. While inter-domain transfers do occur between Archaea and Bacteria, they are less frequent than bacterial-domain confined HGT events (39, 40). Gene exchange between Patescibacteria and their archaeal ultra-small counterparts (DPANN) has been reported (41), particularly in the context of membrane-associated protein-encoding genes potentially involved in host-symbiont interactions (42). Accordingly, we detected nine HT events between Patescibacteria and DPANN archaea (Micrarchaeota and Nanoarchaeota; function of transferred genes discussed below), where Patescibacteria were the predicted donors according to MetaCHIP. A further two genes (one ABC-transporter and one gene related to the BRO family, of which the function remains unknown) were predicted to have been transferred from Nanoarchaeota to Patescibacteria (Table S10). We further investigated the ABC transporter genes of the predicted recipient (Patescibacteria nzgw415) and donor (Nanoarchaeota nzgw43) via phylogenetic analysis. This analysis indicated that it is more likely that Patescibacteria nzgw415, or another Patescibacteria, was the donor of the ABC transporter gene in Nanoarchaeota nzgw43. In addition, homologs of these genes in Patescibacteria and Nanobacteria were found to commonly co-cluster (COG1131, Fig. S5), suggesting that these genes were more widely shared between the two phyla.

### Metabolic functions encoded in horizontally acquired regions

**Fig 6:**
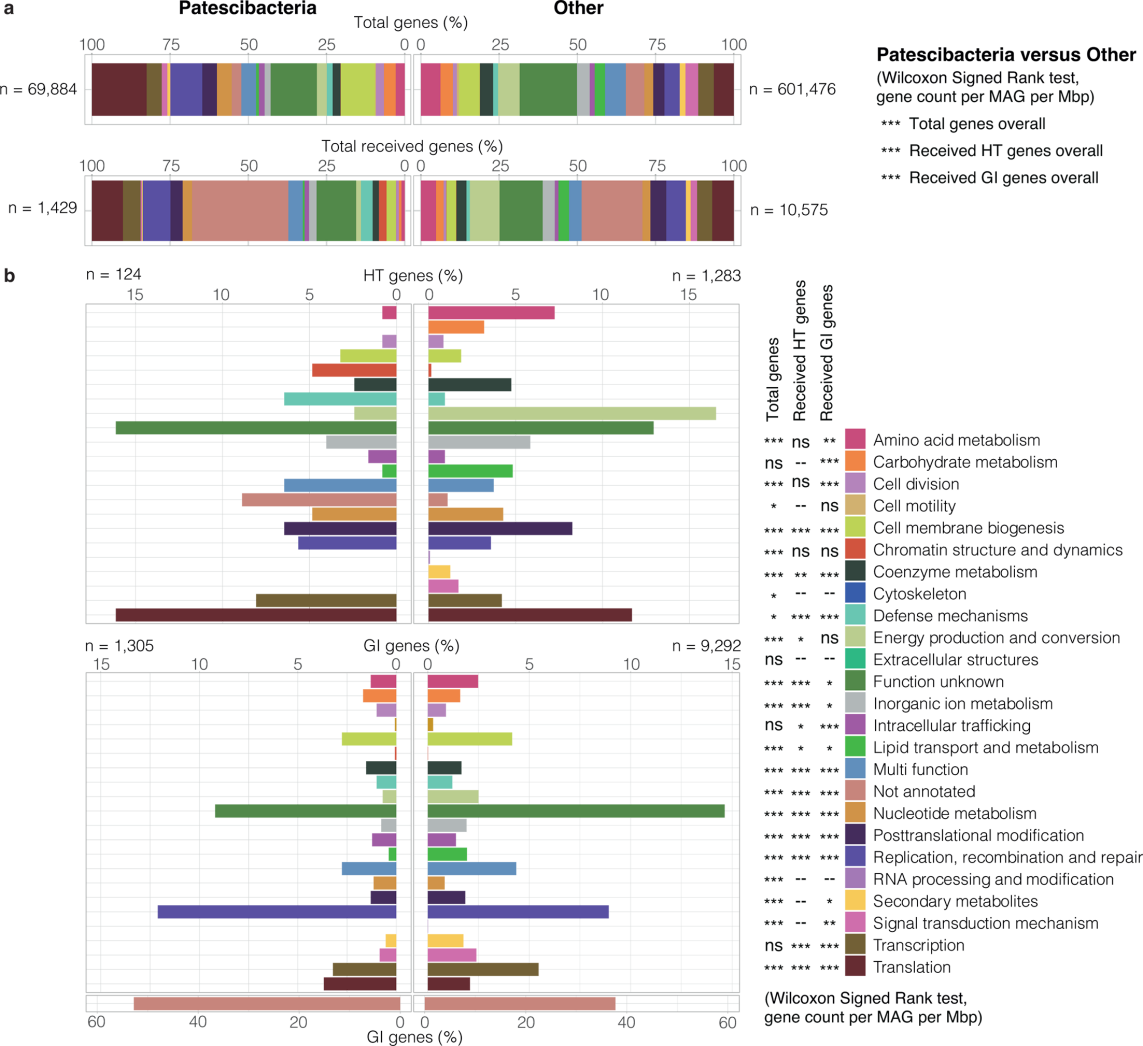
Metabolic functions of genes received through horizontal transfer. (**a**) Metabolic functions encoded by (top) and acquired through HGT (HT and GI genes; bottom) by Patescibacteria (124 HT genes + 120 GIs received) and other groundwater microorganisms (1,283 HT genes + 926 GIs received) from any other member of the groundwater community. (**b**) Breakdown of metabolic functions of HT genes (top) and genes located on GIs (bottom). For (**a**) and (**b**), metabolic functions were assigned using EggNOG categories. Wilcoxon Signed Rank test results are shown for comparisons between Patescibacteria and other taxa for each metabolic category (for genes or HT/GI genes received per MAG per Mbp). *P*-values are ns: not significant, * <0.05, ** <0.01, and *** <0.001. Details are shown in Fig. S6.

Patescibacteria, or CPR bacteria, have reduced genomes and limited biosynthetic capacities (6). The metabolic function of horizontally transferred genes that are retained in Patescibacteria genomes could provide clues about the evolution of dependencies in these ultra-small prokaryotes regarding their symbiotic interactions with hosts. The overall number of genes identified as acquired by Patescibacteria was relatively small (124 HT genes and 1,305 genes in 120 GIs), and a substantial portion of genes located in horizontally acquired genomic regions could not be assigned any function (61.6% of all genes for Patescibacteria, 38.9% for other taxa). However, genes located in horizontally acquired regions in Patescibacteria genomes that were able to be annotated are involved in a wide range of metabolic pathways (Fig. 6).

The predicted HT gene fraction of Patescibacteria was highly enriched, compared to other phyla, in genes assigned to the EggNOG “Defense mechanisms” category (Fig. 6). HT, along with GI genes associated with defense, also exhibited a higher occurrence in Patescibacteria per Mbp (Wilcoxon Signed Rank *P* <0.001), consistent with a slightly higher occurrence of genes overall with defense-associated annotations in Patescibacteria genomes (*P* <0.05, Fig. S6). These included genes (e.g., *macB* and *msbA*) encoding ABC-type systems for multidrug and antimicrobial peptide transport. Membrane-located multidrug transporters have been identified previously in relatives of the ultra-small bacterium *Babela massiliensis* (Dependentiae phylum, previously Candidate division TM6). Their specialization is thought to be important at the host-symbiont interface (42). There is growing evidence that drug transporters are required for the survival of intracellular pathogenic bacteria in host cells (43). Genes assigned to chromatin structure were also more prevalent in the HT gene pool in Patescibacteria (Fig. 6), although they represent a small portion of genes present in both Patescibacteria and other taxa (Fig. 6; Fig. S6). Among these genes were six SWIB/MDM2 domain-containing proteins, which are chromatin-remodeling proteins involved in transcriptional activation (44), and were almost exclusively transferred between Patescibacteria (Table S10). DNA packaging in the form of chromatin is well documented in Eukaryotes and Archaea but remains poorly understood in Bacteria (45). Whether ultra-small bacteria use chromatin-remodeling proteins to manipulate their genomic DNA structure, or for the modification of eukaryotic/archaeal symbiotic host DNA, is unclear.

HT genes involved in translation and transcription were more enriched in Patescibacteria versus other phyla when considering both the acquired gene fraction (Fig. 6) and occurrence per MAG (Wilcoxon Signed Rank *P* <0.001, Fig. 6; Fig. S6). Of these functional categories, Patescibacteria genomes also had significantly more genes in genomic islands and genes overall annotated for translation, but not transcription (*P* <0.001, Fig. S6). Horizontally acquired translation and transcription genes predominantly comprised ribosomal proteins (small and large subunits), elongation factor *tuf*, which plays a central role in protein synthesis, and chaperonin *groS* (Table S10). This was similar when also considering the most frequently transferred genes from Patescibacteria to other groundwater prokaryotes, which notably included the *rpsL* gene encoding ribosomal protein S12 and transcription initiation factor *infA*, alongside translation elongation factor *tuf*. Studies assessing functional categories of horizontally transferred genes have reported widely diverging results with respect to transcription and translation genes. For example, Kanhere and colleagues (46) inferred HGT events based on evolutionary distances within orthologous protein families (using the COG database) and found that genes exchanged among bacteria were primarily involved in translation (46). The “complexity hypothesis” instead suggests genes involved in both transcription and translation are rarely transferred (47). Whereas according to the revisited “complexity hypothesis,” it is only translation-associated genes that are transferred and retained less often, due to a tendency for higher connectedness to other genes, and consequently, a higher number of protein-protein interactions (48). Findings by Oliveira et al. (49) agree with this, showing genes involved in defense, transcription, replication, and repair were those most commonly transferred, and those involved in translation and post-translational modification were the least transferred. Here, the higher proportion of translation genes transferred among and from Patescibacteria may be explained by the overall higher proportion of genes encoding this functional category in Patescibacteria genomes (Fig. 6), alongside the tendency for Patescibacteria to transfer genes to one another (Fig. 4a).

In contrast to Patescibacteria, other groundwater taxa more frequently acquired HT and GI genes with annotations for energy production (respiratory chain) and amino acid biosynthesis (Fig. 6) and had more genes overall associated with these functional categories in their genomes (Wilcoxon Signed Rank *P* <0.001, Fig. 6; Fig. S6). Patescibacteria are known to consistently lack genes for these functions (6) and are likely to scavenge resources such as protons and amino acids from the host or surrounding environment. They are therefore also less likely to transfer genes involved in energy production and amino acid biosynthesis to one another or retain them. Although Patescibacteria received relatively few genes related to energy production and amino acid biosynthesis through horizontal gene transfer (only 11 HT and 18 GI genes), these genes made up a similar or even larger portion of their genomes (per Mbp; Fig. S6) compared to other taxa. This suggests that, despite the low number of such gene transfers, horizontal gene transfer may still play an important role in maintaining and sharing genes associated with energy production and amino acid biosynthesis within Patescibacteria. A higher proportion of genes received by other taxa was further predicted to be involved in secondary metabolite pathways and signal transduction mechanisms (426 HT and GI genes acquired by 105 MAGs affiliated with other phyla versus 18 GI genes in 15 Patescibacteria MAGs) (Fig. 6; Tables S10 and S11), and genes with these functions were again more prevalent in the genomes of other groundwater taxa (Wilcoxon Signed Rank *P* <0.001, Fig. S6). Secondary metabolites are considered luxury metabolism (50) and can constitute public goods (e.g., siderophores) (51). As such, biosynthetic gene clusters encoding these metabolites are uncommon in streamlined genomes (52, 53), which have reduced auxiliary functions (54), and are more likely to depend on the metabolites produced by other organisms (55). Similarly, loss of signal transduction mechanisms, which facilitate environmental monitoring, is a characteristic feature of reduced or streamlined bacterial genomes, including those of bacteria with non-intracellular lifestyles (e.g., bacterioplankton) (56, 57).

The extent to which gene function impacts HGT is uncertain. Previous studies suggest that gene function either appreciably impacts HGT (46, 49, 58) or is insignificant (48, 59). Moreover, reported biases in horizontally transferred gene functions are varied and include, for example, the enrichment of cell surface, DNA binding, and pathogenicity-related functions (58), enrichment of defense, transcription, replication, and repair-related functions (49), or the enrichment or depletion of translation-associated functions, as discussed above. Here, when considering the type of gene transfer, we found in general that genes localized in GIs exhibited a more equivalent distribution of metabolic functions between Patescibacteria and the rest of the groundwater community compared with individually transferred genes (Fig. 6). Despite this, the difference in genomic composition between the two taxa groups resulted in both HT and also GI types contributing significantly different proportions of genes across several functional categories to the genomes of Patescibacteria versus other taxa. Results therefore suggest that the lifestyle of the recipient impacts the traits of genes transferred and also retained, and their contribution to the genome as a whole. Biases toward horizontally acquired features distinct from the general groundwater communities by Patescibacteria may be explained by constraints on streamlined genomes. Selection of distinct features could occur by two mechanisms: (i) retention of horizontally transferred genes and concentration due to selective deletion of other less desirable features and (ii) selective retention of acquired genes with desirable features. Caveats are that the distribution of functions among the hypothetical coding DNA sequence (CDS) fraction in Patescibacteria is unknown, and that confirmation is needed from further studies, given the small sample sizes of HT genes per category.

Given the apparent difference in gene functions between individually transferred genes and GIs (Fig. 6), we questioned whether the length of transferred DNA was an important factor affecting integration and retention of transferred genes and related metabolic functions in streamlined ultra-small and other groundwater prokaryotes. The length of transferred DNA was 10,324 ± 9,397 bp on average for the 1,046 detected GIs (10.8 Mbp total in all groundwater taxa) and 899 ± 562 bp for the 1,487 HT genes (1.3 Mbp overall) (Table S6). Therefore, while the number of GIs detected was a third less than for individually transferred genes, the total length of the GI sequence transferred was substantially greater. GI length was also broadly equivalent between Patescibacteria (9,689 bp per MAG) and other groundwater microorganisms (10,406 bp per MAG) (albeit slightly higher proportionally in Patescibacteria, Fig. 1c), implying that GIs are an important source of new functions among Patescibacteria and diverse groundwater microorganisms. Besides sequence length, it has been shown that recombination efficiency decreases exponentially with sequence divergence (28, 60). However, the presence of flanking regions of identity in exchangeable regions can remove most barriers to recombination. The shortest length of sequence homology necessary for efficient recombination can vary greatly depending on the organism, the recombination pathway used, and other factors (61). In *Escherichia coli*, for example, efficient recombination has been observed with as little as 23 bp of sequence homology (62).

### Impact of phylogenetic distance and direction of transfer

**Fig 7:**
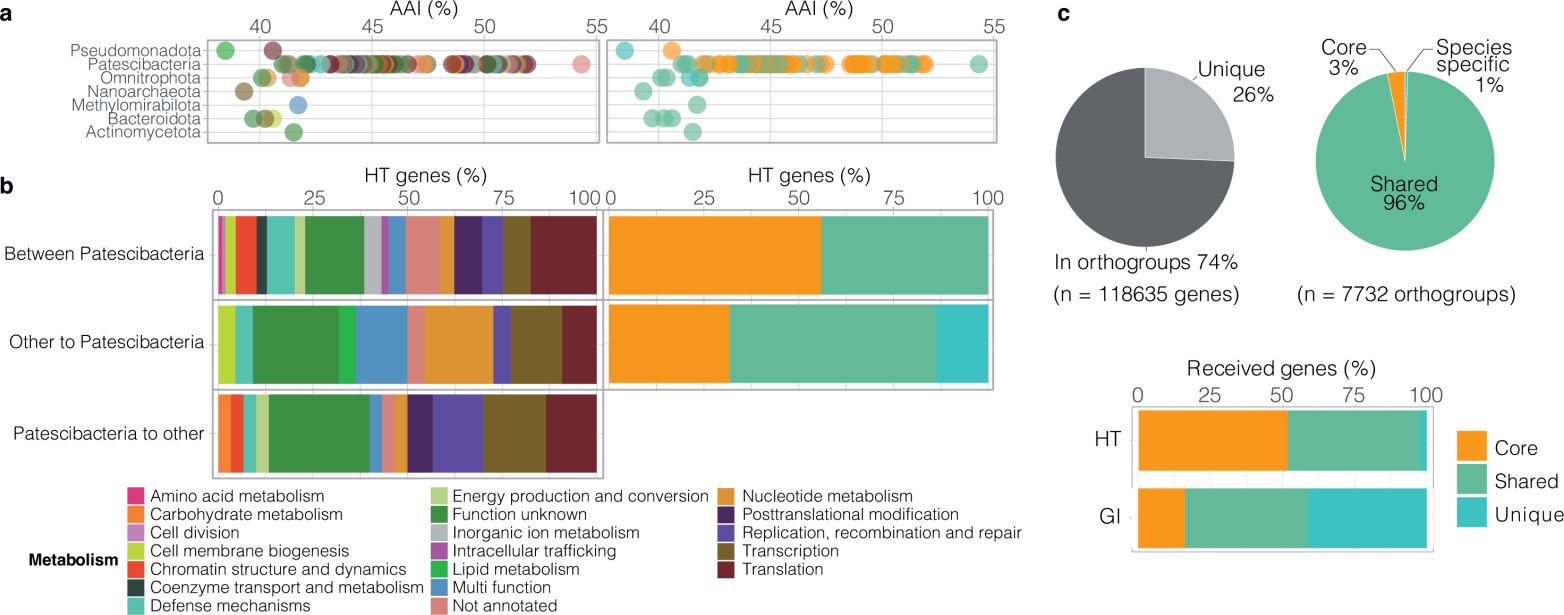
Pangenome of Patescibacteria and effect of phylogenetic distance and direction of transfer on acquired genes. (**a**) Impact of phylogenetic distance of HGT donor to Patescibacteria (calculated as AAI) on metabolic function of horizontally acquired genes (left) and pangenome category (right). (**b**) Metabolic function of HT genes according to direction of transfer. (**c**) Proportion of Patescibacteria total gene pool contained in orthogroups vs. unique, and breakdown of proportion of orthogroups in “core”, “shared” and “species-specific” pangenome categories (top). Proportion of horizontally acquired genes (HT genes and GI genes) by Patescibacteria taxa in each pangenome category (bottom).

We assessed how the phylogenetic distance between putative donor-recipient pairs associates with the metabolic function of genes acquired by Patescibacteria. Genetic exchange occurred over a wide range of phylogenetic distances based on average amino acid identities between genomes of groundwater microorganisms (as low as 38.5% AAI, Fig. 7a). Within the Patescibacteria phylum, genes of diverse metabolic functions were transferred regardless of phylogenetic distance (41.0%–54.3% AAI). However, the metabolic function of horizontally transferred genes appeared to depend on the predicted direction of HT events (Fig. 7b). For HT events within the Patescibacteria phylum, a small number of metabolic functions were found to be exclusively transferred among Patescibacteria: annotations indicate these functions (encoded by 12 out of 104 HT genes shared between Patescibacteria) were involved in processes such as amino acid metabolism (serine hydroxymethyltransferase, GlyA), cell division (actin homolog, MreB), intracellular trafficking, coenzyme metabolism (riboflavin biosynthesis), and inorganic ion metabolism (cation transport) (Fig. 7b; Table S10).

For HT events predicted to be from Patescibacteria to other groundwater microorganisms, other functions were exclusively transferred (out of *n* = 29 transfers overall, Fig. 7b), namely two genes associated with chromatin structure and dynamics (SWIB/MDM2 domain-containing protein) and carbohydrate metabolism (mannose-6-phosphate isomerase, COG0662). Moreover, Patescibacteria were predicted to have donated a greater fraction of replication, recombination, and repair genes (GIY-YIG catalytic domain-containing protein, UvrD subfamily helicase, bacterial nucleoid DNA-binding protein COG0776, DNA-3-methyladenine glycosylase) to other bacteria. Among the 10 putative HT events from Patescibacteria to DPANN archaea reported above (including the ABC transporter gene), three were related to a glyoxalase (Table S10), which is a key metalloenzyme in the glycolytic pathway involved in the detoxification of reactive methylglyoxal into D-lactate. It is a common feature across domains of life, including in Archaea (63), and was previously reported in single cell amplified CPR genomes (40). The reason ultra-small archaea exhibit an apparent preference for acquiring certain bacterial homologs is unclear.

For horizontal gene transfer predicated as occurring from other prokaryotes to Patescibacteria, there is evidence of a very small number of HT events that occurred exclusively (2 out of 20 transfers overall, Fig. 7b). Annotations indicate these comprised the *acpP* gene, encoding an acyl carrier protein, and a gene encoding a putative lysophospholipase (COG2267, alpha beta hydrolase superfamily). The lysophospholipase gene was identified as a transfer from a member of the Pseudomonadota (MAG nzgw525) to the Paceibacteria MAG nzgw447 by MetaCHIP. Further phylogenetic analysis supported this prediction and showed close clustering of recipient and donor genes (Fig. S7). Lysophospholipases catalyze phospholipid hydrolysis and generate fatty acids. A recent study coupling lipidomics and metagenomics approaches suggested that members of the Patescibacteria phylum that lack capacities for fatty acid biosynthesis (6) can recycle lipids from other bacteria for cell membrane biogenesis (64). Results here further suggest that HGT could facilitate the sourcing of fatty acids by Patescibacteria. Phospholipase genes have also been reported in the genomes of other ultra-small CPR prokaryotes (e.g., in amoeba endosymbiont *Vampirococcus lugosii*; [65]). Patescibacteria were also more likely to receive genes from other groundwater microorganisms associated with nucleotide metabolism (*n* = 2), including one putative thymidylate synthase gene *thyA*. The encoded enzyme catalyzes the *de novo* synthesis of dTMP (deoxythymidine monophosphate), an essential precursor for DNA biosynthesis (66), which is a capacity already encoded by the minimal biosynthetic gene repertoire of groundwater Patescibacteria. Results indicate that this essential function could also be horizontally acquired from other members of groundwater microbial communities.

While the number of transfer events recorded from general groundwater microorganisms to Patescibacteria and vice versa is low, limiting robust conclusions, these numbers are consistent with known associations between HGT and phylogenetic distance among prokaryotes more generally (28). Many metabolic functions gained via HGT in ultra-small prokaryotes remain poorly studied, regardless of phylogenetic origin. However, where annotations are available events identified here tend to confer Patescibacteria with critical functions, such as the ability to degrade lipids and carry out the biosynthesis of nucleotides.

### Patescibacteria pangenome and HGT

The evolutionary fate of an HGT event is determined by the fitness conferred to the recipient genome and cell, that is, whether the acquisition of exogenous DNA is beneficial, neutral, or deleterious. To evaluate the importance of the metabolic functions predicted to be acquired through HGT in Patescibacteria, we performed a pangenome analysis of the 125 reconstructed MAGs from the 16 groundwater samples in this study (notably comprising members of the classes Paceibacteria, ABY1, and Microgenomatia, Fig. 1a). Genes in the pangenome were classified into three groups based on their occurrence: (1) the “core” genes, which are present in at least 2/3 of all 132 genomes (2), the “shared” genes are found in more than one genome, but are not core genes, and (3) the “unique” genes, comprising species-specific genes not assigned to an orthogroup (i.e., genes present in one genome only). Both shared and unique genes comprise the accessory genome. The pangenome of all the examined Patescibacteria contained 7,732 orthogroups, comprising 88,187 out of the total 118,635 predicted genes (i.e., 74.3% of genes had orthologs in two or more genomes). Results indicated the vast majority of orthogroups (96%, 7,449 orthogroups comprising 56,524 genes) were “shared” between the groundwater Patescibacteria, and only 3% of orthogroups (247 orthogroups comprising 2,634 genes) comprised the core genome (Fig. 7c). The small core is expected given the pangenome was undertaken at the phylum level, and as members of the CPR radiation (Patescibacteria) have been shown by Méheust and colleagues to possess a smaller, albeit distinctive, set of core protein families compared to other bacteria (67).

As also indicated by results presented above on metabolic functions encoded in horizontally acquired regions, GIs brought many unique, and hence divergent, genes to the Patescibacteria pangenome (41.2% of GI genes, Fig. 7c). These observations are consistent with previous work suggesting that prokaryotes contain a higher proportion of novel genes in GIs compared with the rest of their genome (68). HT genes putatively acquired by Patescibacteria species were spread across the core and the shared gene fractions of these organisms (45.8% shared and 51.9% core, Fig. 7c), and not the unique fraction. A high proportion of core or shared genes would be expected given predicted transfers occurred mostly between Patescibacteria species (83.2% of HT events, Fig. 4a). The lack of any unique HT genes detected likely reflects both the rarity of relatively recent transfers from other bacteria to Patescibacteria (such that they are found in a single patescibacterial representative) alongside non-recovery of the donor taxa in the data set (HT events by the MetaCHIP tool require a donor to be present) (17).

Streamlined genomes are characterized by low numbers of paralogs (54). Accordingly, among all genes in Patescibacteria, we detected a low number of paralogous orthogroups based on the pangenome analysis results (89 genes in 36 species-specific orthogroups—that is, 89 paralogs out of 118,635 genes). We found that neither genes located on GI regions nor HT genes were paralogs of other genes in the Patescibacteria pangenome. Gene duplicates are generally under purifying selection (69) and have been shown to evolve faster than non-duplicated genes with a similar level of divergence (70), introducing new proteins. Nonetheless, gene duplication is usually widespread in bacterial genomes, with an estimated 7%–41% of bacterial proteins encoded by paralogs (71), and is a common strategy for creating novel gene functions for niche expansion in prokaryotes (72). However, it has been quantitatively demonstrated that gene gain via HGT rather than gene duplication is the principal factor of innovation in the evolution of prokaryotes (73). Based on a comparably high proportion of HGT into Patescibacteria as for other phyla, results here demonstrate HGT and unique genes conferred by GIs are almost exclusively used over duplication by Patescibacteria to expand niche ranges.

### GI in Patescibacteria nzgw456 encoding a full type I restriction-modification system

**Fig 8:**
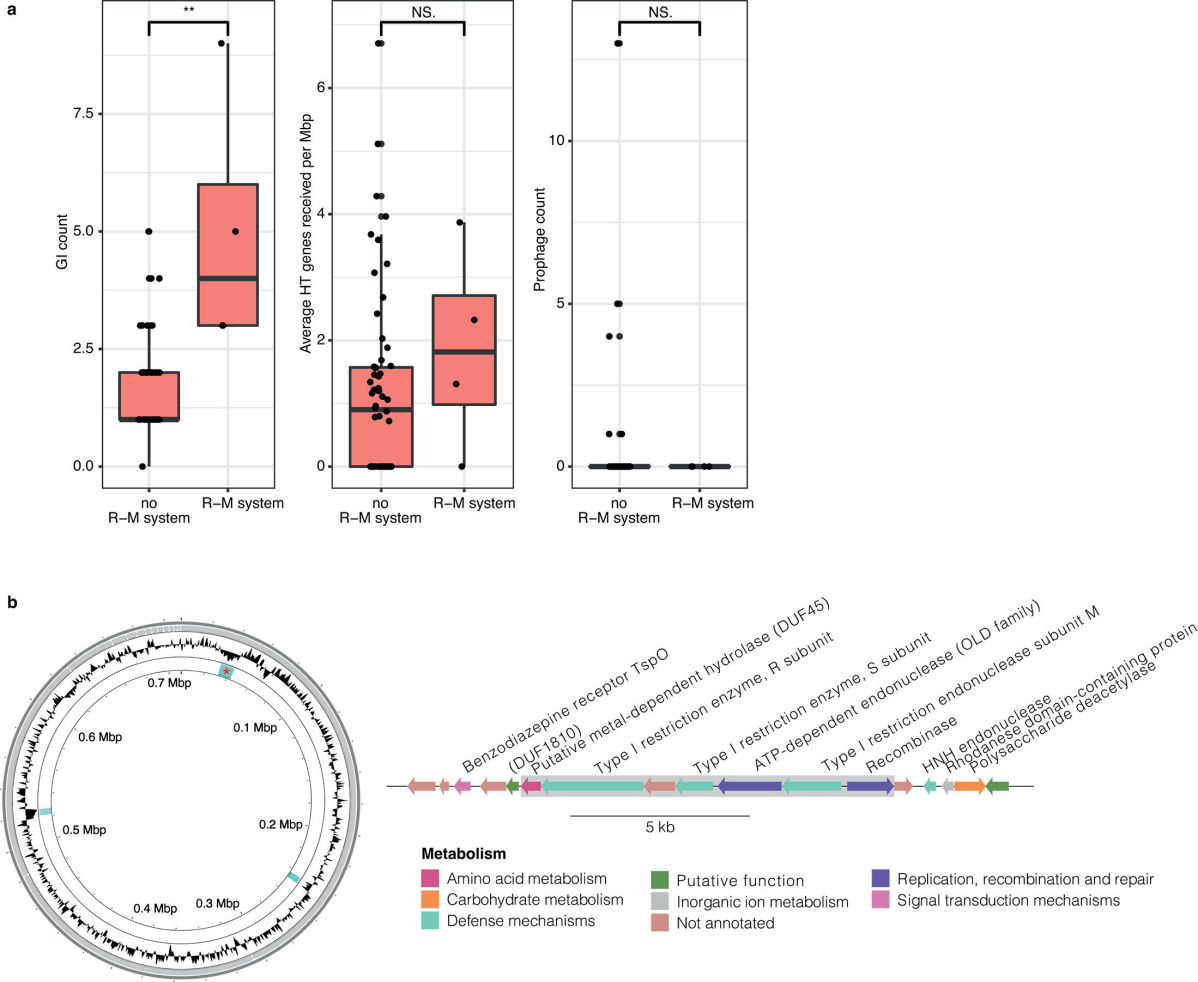
Association of restriction-modification systems and HGT. (**a**) GI, HT, and prophage counts based on presence or absence of an R-M system in each patescibacterial MAG in our data set, and any R-M system detected anywhere in the genome. The center line of each boxplot represents the median; the top and bottom lines are the first and third quartiles, respectively; and the whiskers show 1.5 × the interquartile range. Significant differences were assessed for each group using Wilcoxon Signed Rank (**: *P* < 0.01). (**b**) Genomic island encoding a full type I restriction-modification system in Patescibacteria nzgw456. Left: circular genome plot with the following rings (inner to outer): GIs (in blue; star indicates location of GI depicted in the right panel), GC content, and contig boundaries. Right: Linear map of GI (indicated by gray box) and protein-coding genes. Further examination of the GI was undertaken to confirm boundaries using the DarkHorse prediction tool (77).

Bacteria (and archaea) with reduced genome sizes have been hypothesized to be largely lacking classical defense mechanisms, such as CRISPR-Cas (Clustered Regularly Interspaced Short Palindromic Repeats—CRISPR associated), restriction-modification (R-M), and toxin-antitoxin systems (74). Similarly, the analysis of hundreds of CPR/Patescibacteria genomes recovered from diverse environments revealed that most are missing CRISPR-Cas adaptive immune systems, lost in the process of genome streamlining, and instead rely on nonspecific defense, such as R-M systems (6). Although first believed to constitute a barrier to genetic exchange between organisms (75), it has been recently suggested that these systems play an important role in HGT rates by increasing the frequency of exchange in organisms possessing numerous R-M systems (76). The four patescibacterial MAGs in this study with R-M systems annotated had significantly more GIs than other patescibacterial MAGs, although they did not have more HT genes or prophage (Fig. 8a). While the small sample size (*n* = 4) limits our ability to conclude any clear relationship between HGT frequency and R-M systems, we found no evidence that R-M systems appreciably inhibit genetic exchange overall. Further studies are needed to determine the relationship between GI frequency and R-M systems in Patescibacteria.

Some R-M systems were found to be horizontally acquired by Patescibacteria. One GI in Patescibacteria nzgw456 (Paceibacteria class) was found to encode a full type I R-M system (Fig. 8b). Type I R-M systems, which are the most complex of all four known R-M systems (78), comprise three subunits, namely HsdR and HsdM (required for methyltransferase activity), and HsdS (required for restriction). Co-located with genes encoding these subunits on the GI was one putative ATP-dependent endonuclease. Type I R-M-related genes were also found in a GI present in Patescibacteria nzgw350, and other restriction endonucleases in nzgw410 and nzgw504 (Table S12). Acquisition of type I R-M genes via HGT was recently described in the CPR bacterium *V. lugosii* (65), however, not as syntenic genes. It has been suggested that R-M systems could be used by putative epibionts as a way to source nucleotides by degrading exogenous DNA, including DNA derived from viruses (79), in addition to protecting against phage infection (80).

### Conclusions

Recent phylogenetic analyses have shed light on the massive bacterial group that is the Patescibacteria phylum/CPR. Due to the reductive evolution of Patescibacteria, it is essential to consider the processes of gene gain and loss alongside the predicted symbiotic lifestyles of these ultra-small microorganisms. Results here indicate that Patescibacteria are involved in dynamic genetic exchange with their ultra-small counterparts and other members of the microbial community, and that gene exchange rather than duplication is the primary means of genome innovation in these bacteria. We found a total of 124 HT events involving Patescibacteria (which is likely to be an underestimate), 10 prophages, and over 100 GIs in Patescibacteria genomes.

Gene acquisition by Patescibacteria likely occurs via multiple mechanisms (conjugation, natural competence, and transduction). Genes encoding pili and competence are prevalent among Patescibacteria and are proposed to facilitate DNA uptake (6, 11), which our findings support. Viral predation is potentially also an important mechanism of gene exchange for Patescibacteria, given that they exhibit a similar capacity for predation as other bacteria in groundwater (22). Regardless of the mechanism, gene exchange requires close proximity. Given typically low concentrations of planktonic biomass in groundwater, most gene exchange is probably facilitated by the proximity afforded by biofilm colonization (findings indicate a sizeable fraction of ultra-small taxa in alluvial aquifers is sediment attached; [16]) and/or an epibiotic lifestyle. Evidence suggests Patescibacteria form epibiotic associations with hosts in groundwater (7, 81), which would afford close proximity, not only to the host, but among the host-attached cells (82, 83).

While most of the gene transfers identified in this study occurred among Patescibacteria, predicted HGT origins were diverse and widespread across both bacterial and archaeal domains, and indicated interactions with members of the Omnitrophota and Bacteroidota phyla. Predicted functions encoded by horizontally acquired regions comprised diverse pathways, but mainly transcription, translation, and DNA replication, recombination, and repair, such as ribosomal proteins and transcription factors. Findings show that acquired genes were distributed across core and accessory portions of Patescibacteria genomes, and that GIs generally introduced phylogenetically ambiguous and novel sequences. Some of the metabolic functions horizontally acquired by Patescibacteria are distinct from those acquired by the general groundwater communities, suggesting unique pressures on gene gain/loss dynamics occurring in ultra-small prokaryotes.

## MATERIALS AND METHODS

### Sample collection

Groundwater samples were collected from eight wells from two sand/gravel aquifers in Canterbury, New Zealand, as described by (84). Briefly, wells were purged (×3–5 bore volumes), then 3–90 L of groundwater was filtered per sample. Biomass was captured on 0.22 µm mixed cellulose ester (MCE) filters, after passing through a 1.2 µm MCE pre-filter, using a 142 mm filter holder (Merck Millipore Ltd., Cork, Ireland). Samples were preserved in RNAlater (ThermoFisher Scientific, Waltham, MA, USA), transported on dry ice, and stored at −80°C.

After collecting a groundwater sample (as above), a second sample per well was collected following low-frequency sonication (2.43 kW for 2 minutes) to induce biofilm (and sediment) detachment from the surrounding aquifer, using a custom sonication device (85). 0.5–15 L of sediment (and hence biomass) enriched groundwater was filtered as above.

### Nucleic acid extraction and amplicon sequencing

To remove RNAlater, filters were centrifuged (2,500 g for 5 min) and washed with nuclease-free phosphate-buffered saline (pH 7.4, ThermoFisher Scientific, Waltham, MA, USA). DNA was extracted using the DNeasy PowerSoil Pro Kit (Qiagen, Valencia, CA, USA) and 0.25–0.9 g of whole filter per reaction. Samples were extracted in replicate (1–47 reactions/sample). Replicates were pooled and concentrated using a sodium acetate/ethanol precipitation with glycogen (0.1 µg/µL final concentration, Roche, Basel, Switzerland). High molecular weight DNA was confirmed via gel electrophoresis. DNA was quantified using Qubit fluorometry (ThermoFisher Scientific), and quality was checked using a NanoPhotometer (Implen, Munich, Germany). Extractions yielded 70–864 ng of DNA (8.7 ng for sample gwj02) for metagenomics. 16S rRNA gene amplicons were generated from the 16 groundwater samples, amplicon sequence variants determined, and taxonomy assigned as previously described (16).

### Metagenome sequencing

Whole genome shotgun sequencing was undertaken on the 16 Canterbury samples (gwj01-16). DNA libraries were prepared using the Illumina TruSeq Nano DNA Kit with ~550 bp inserts, by Otago Genomics (University of Otago, Dunedin, NZ). The low-yield gwj02 sample was prepared with the ThruPLEX DNA-Seq Kit (Takara Bio USA, Inc., Mountain View, CA, USA). Sequences (2 × 250 bp) were generated using the Illumina HiSeq 2500 V4 platform. Adapter sequences were removed using Cutadapt v2.10 (86), and reads were quality trimmed using sickle v01.33 (parameters -q 30 L 80) (87). Read quality was checked using FastQC v0.11.7 (https://www.bioinformatics.babraham.ac.uk/projects/fastqc/).

### Metagenome assembly and binning

The 16 metagenomes were individually assembled using SPAdes v3.11.1 (88) (parameters: --meta, -k 43,55,77,99,121). Metagenomes from the same well were also co-assembled (same parameters). A dereplicated set of MAGs (99% average nucleotide identity, ANI, dereplication threshold) was generated and refined as described previously (84). Refinement involved assessment of contig clusters associated with bins using t-distributed stochastic neighbor embedding transformation of contig tetranucleotide frequencies and coverage to remove poorly clustered contigs and for cluster visualization (https://github.com/dwwaite/bin_detangling).

For ultra-small prokaryotes, genome completeness and contamination were assessed based on the presence of the following SCG sets: 43 SCGs for Patescibacteria (5), 51 bacterial single-copy genes (SCGs) for Dependentiae (89), and 38 archaeal SCGs for DPANN (89), using the method described by Brown et al. (5). For other prokaryotes, completeness was estimated using SCG analysis with CheckM v1.0.13 (90). MAGs were retained for gene exchange analysis if ≥70% complete and <5% contaminated based on custom SCG analysis for ultra-small taxa and CheckM analysis for other taxa. For comparison, MAG completeness estimates were also subsequently determined using the machine learning approach employed by CheckM2 v1.0.1, which has been shown to accurately predict genome completeness in ultra-small prokaryotes (91). Pearson correlation of custom SCG and CheckM2 completeness estimates for ultra-small taxa indicated a high level of agreement between methods (*r* = 0.63, *P*-value <2.2e-16). Completeness and contamination estimates are given in Table S1 and compared in Fig. S8). To determine coverage, reads were mapped onto the de­replicated MAGs using Bowtie (92). Sample-specific genome relative abundance was calculated by normalizing based on the highest read count between samples (36).

### Gene prediction and functional annotation

Protein coding sequences were predicted using Prodigal v2.6.3 (93). Genes were annotated using USEARCH (94) against UniRef100 (95), Uniprot (96), and KEGG (97) databases (e-value ≤0.001 and identity ≥50%), and HMMs against PFAM (98) and TIGRFAM (99) databases using HMMER v3.1b2 (100) (e-value ≤0.001). In addition, functional annotation was carried out using eggNOG-mapper v2 (101) against the eggNOG v5.0 database (102), using default parameters.

### Genome taxonomy and phylogenetic tree

Taxonomic classification of MAGs was performed using the Genome Taxonomy Database Toolkit (GTDB-Tk, v2.1.1) (103) with database release 214 (104). Maximum likelihood trees were constructed using 120 bacterial and 53 archaeal concatenated marker genes with IQ-TREE v1.6.12 (105) using 100 bootstraps, and ModelFinder (106) best-fit model LG + F + G4 for bacteria and LG + F + I + G4 for archaea. Trees were visualized and annotated with iTOL (107). MAGs (≥80% complete) were compared to GTDB representative genomes (downloaded 20 January 2020) by calculating average amino-acid identities (AAI) using BLASTp (108) matches sharing ≥30% identity over ≥70% of alignment length.

### Identification of horizontal gene transfer

Individual HT events among MAGs were identified using MetaCHIP v1.10.0 (17) at phylum, class, order, family, and genus levels. False-positive HT genes (potentially introduced via assembly contamination) were filtered out by removing HT genes marked by MetaCHIP as “full-length match” (where the match represents a large proportion of the contig) or “end match” (HT genes located at the end of the contig) as described by Dong et al. (34). Information about HGT events involving members of the Patescibacteria phylum was retrieved and visualized using the Circlize package in R (109).

For genomic island prediction, contigs were concatenated into contiguous sequences and uploaded to the IslandViewer4 web server (110) for genomic island prediction. GIs that were predicted by at least one method and that did not overlap two concatenated contigs were considered for the analysis. Genes located on GIs were compared to community gene content using BLASTn (108).

Prophages integrated into the Patescibacteria MAGs were identified as described previously (22). Whenever a predicted viral sequence was overlapping a predicted GI, CheckV quality assessment information was used, and only “high-quality” genomes were retained as prophages (111). Functional gene annotations were generated using DRAM-v v1.3.5 (112). Linear genome maps were created using the genoPlotR package (v0.8.11) (113).

### Phylogenetic distance

Average amino acid identities (AAI) between MAGs (≥80% complete) involved in HGT events were determined via BLASTp matches sharing ≥30% identity over ≥50% of query and database alignment length.

### Orthology analysis

To investigate the Patescibacteria pangenome, we performed an orthologous clustering of 118,635 protein-coding sequences encoded by the recovered MAGs. Pairwise all-vs-all BLASTp searches were conducted using the following cut-offs: ≥30% nucleotide identity over ≥50% of query sequence length, e-value ≤0.001. BLAST table outputs were used as input for orthogroup clustering using Orthofinder v2.3.1 (114) with the -b option and default parameters.

### Protein sequence trees

To compare the phylogenetic placement of donor and recipient genes, protein sequence trees were built for four of the predicted transferred genes: LysR (COG0583), DNA topoisomerase (COG0187), ABC transporter (COG1131), and lysophospholipase (COG2267). LysR protein coding sequences (annotated as COG0583, *n* = 1,896) were retrieved from our dataset. Five reference sequences were downloaded from NCBI RefSeq (23 July 2024). The reference sequences include all Omnitrophota sequences found in RefSeq (search “lysR[All Fields] AND (“Candidatus Omnitrophota”[Organism] OR omnitrophota[All Fields]”), and one well-annotated sequence from *Escherichia coli* (NP_417316.1). Only sequences longer than 50 amino acids were considered. Those from our study from phyla (excluding Patescibacteria and Omnitrophota, and the five references) were clustered using usearch v11.0.667 (minimum 30% identity) (94), resulting in 917 clusters. Representative sequences from these clusters, LysR sequences from Patescibacteria and Omnitrophota in this study (*n* = 13, all >50 amino acid long), and the reference *Escherichia coli* sequence (NP_417316.1) were used for building a tree of LysR sequences. The protein sequence alignment was carried out using MUSCLE (v3.8.31) (115) with default settings, and the alignment was trimmed using trimAl (v1.4.1, -automated1 option) (116). The tree was constructed using FastTree with default settings, including 1,000 bootstrap replicates (117). A subset of LysR sequences (56 from this study and the five references) was also aligned as above and was used to recreate the tree using IQ-TREE v2.2.2.2 (105) and ModelFinder (106) best-fit model LG + F + R3 and 1,000 bootstrap replicates. Protein-coding sequences for the remaining three gene trees were retrieved from the MAG data set, and sequences excluding ones of donor/recipient phyla were clustered using usearch as above. Representative sequences were then aligned and trees were built using FastTree as for the LysR tree.

### Diversity and statistical analyses

Analyses were conducted using R v4.2.1. Taxa accumulation and rarefaction curves were generated using vegan v2.6.4 with the specaccum and rarecurve functions and using MAG coverage data (Table S2). MAG prevalence was plotted using the R hist function. Rank abundance curves were generated using the R plot function for the overall MAG data set, and the matplot function for MAGs per sample. Pearson correlations were undertaken using the R cor.test function.

## Data Availability

Sequence data are deposited with NCBI under BioProject PRJNA699054.

## SUPPLEMENTAL MATERIAL

Supplemental figures msystems.00046-25-s0001.pdf Figures S1 to S8.

Supplemental tables msystems.00046-25-s0002.xlsx Tables S1 to S12.

ASM does not own the copyrights to Supplemental Material that may be linked to, or accessed through, an article. The authors have granted ASM a non-exclusive, world-wide license to publish the Supplemental Material files. Please contact the corresponding author directly for reuse.
