# Supplementary material for: Genetic exchange shapes ultra-small Patescibacteria metabolic capacities in the terrestrial subsurface
Source: mSystems. 2025 Aug 15;10(9):e00046-25. doi: 10.1128/msystems.00046-25 (PMC12455944; doi:10.1128/msystems.00046-25)
Supplement: Supplemental figures — Figures S1 to S8. [file msystems.00046-25-s0001.pdf]

Supplementary figures

(Gios et al. 2025 mSystems)

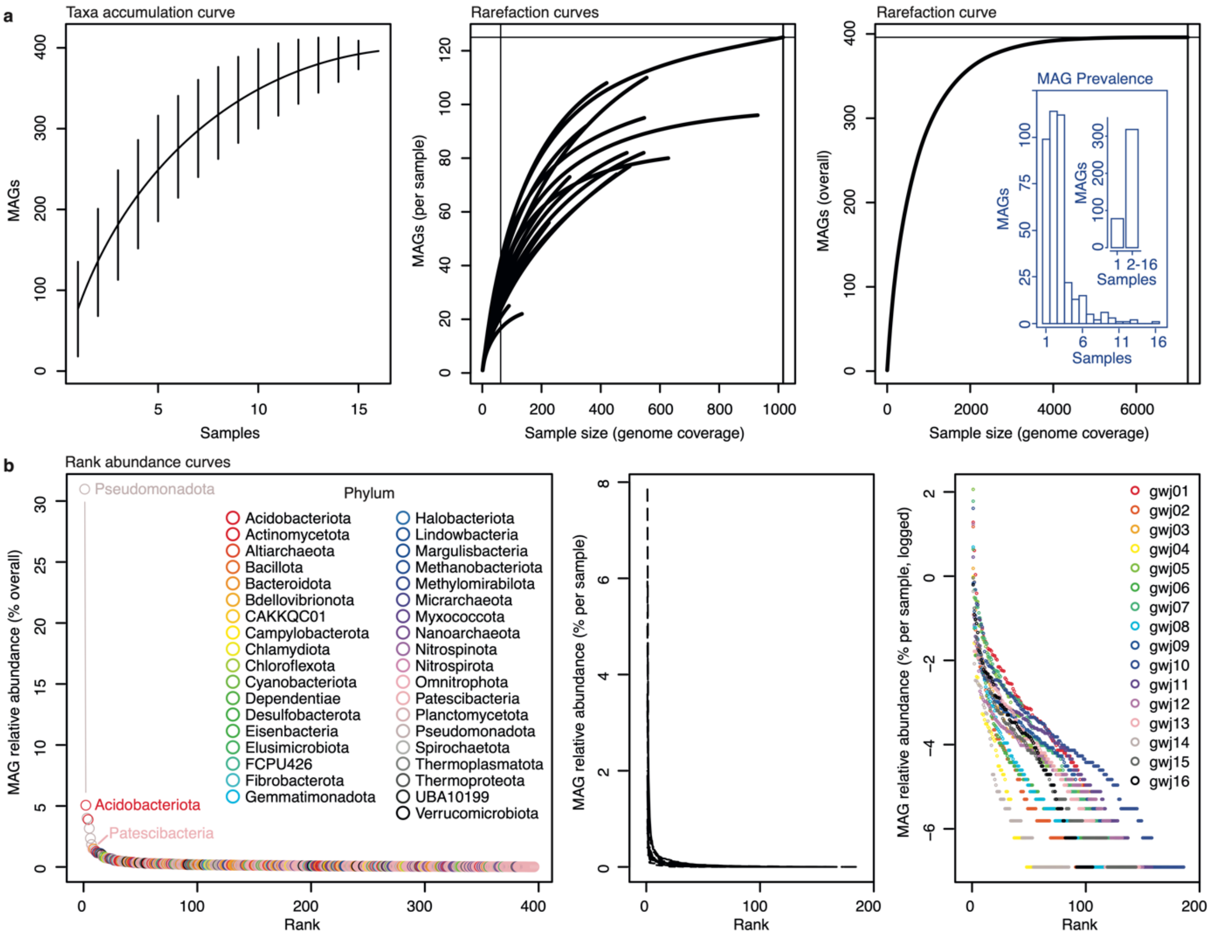

Figure S1. Diversity of MAGs in the non-redundant and quality filtered dataset (n=396). (a) Taxa accumulation curve (leftmost plot) and rarefaction curves for each sample and overall (plots to the right). Plots were generated using MAG coverage data rounded to the nearest whole number. Inset in the rightmost plot in blue shows MAG prevalence across samples with the threshold for presence set at ≥70% genome coverage. (b) Rank abundance curves for MAGs in the dataset overall with MAGs data points coloured by phylum (leftmost plot). Rank abundance curves for MAGs per sample (plots to the right), with MAG data points in the rightmost plot coloured by sample.

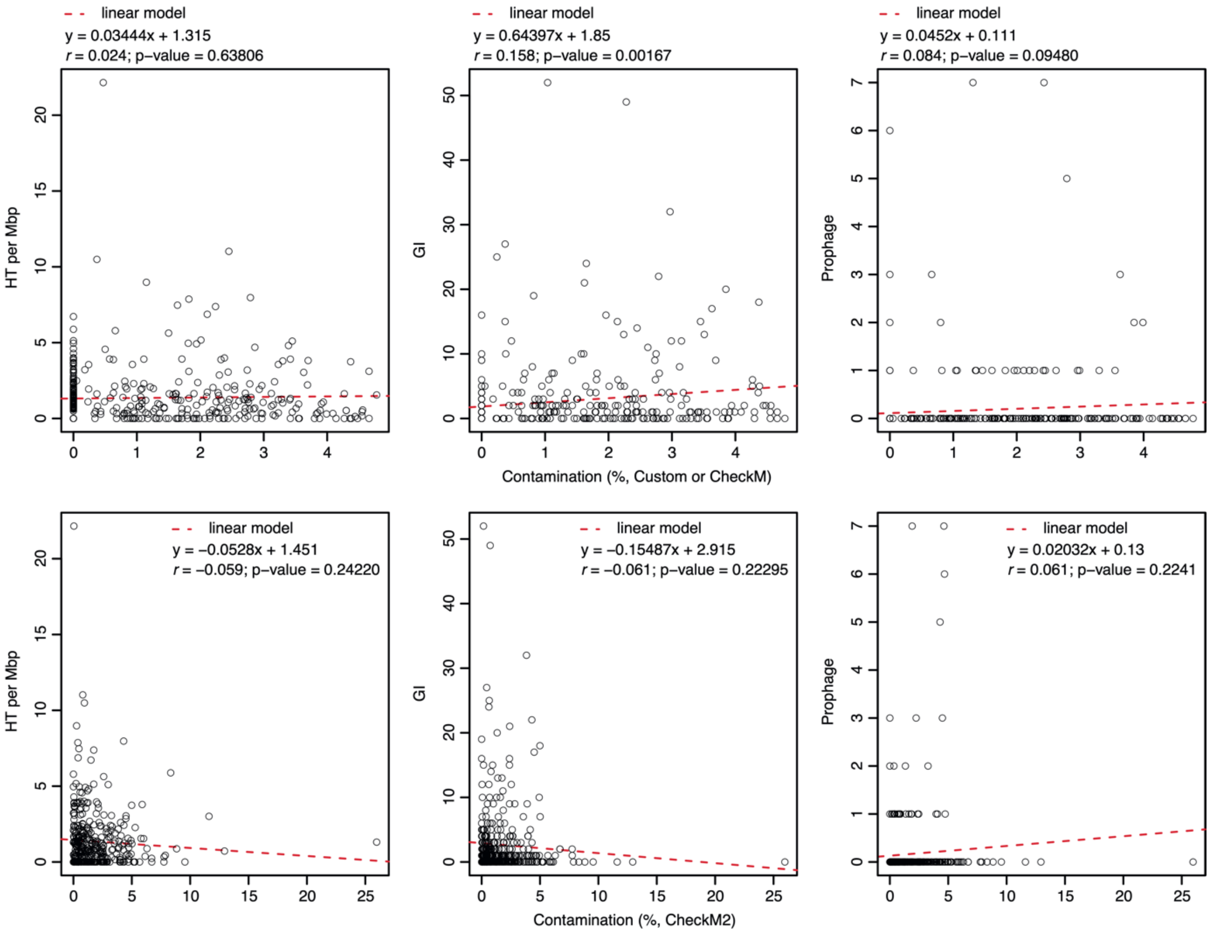

Figure S2. Comparison of MAG contamination estimates versus counts for HT genes, GIs and prophage. Upper and lower plots are based on estimates generated by the SCG (custom or CheckM) and CheckM2 approach, respectively. Linear models are fitted to the data, and Pearson correlations are shown for each plot. Contamination estimates are not correlated with HGT metrics in all cases, except for the comparison between the SCG approach and GI counts for which a weak Pearson correlation was obtained (linear model adjusted $R^2 = 0.02233$). However, no clear trend is observable in the plotted data, and the analogous comparison between CheckM2 estimates and GI counts indicated there was no linear trend. The presence of HT genes (n = 8) was also confirmed in 3 of 5 recipient Patescibacteria MAGs with the highest contamination (nzgw355, 3 genes; nzgw350, 4 genes and nzgw346, 1 gene). Comparisons used MAGs from the same populations (pre-dereplication) that were independently assembled from different samples. The two remaining MAGs are unique (nzgw373 and nzgw432).

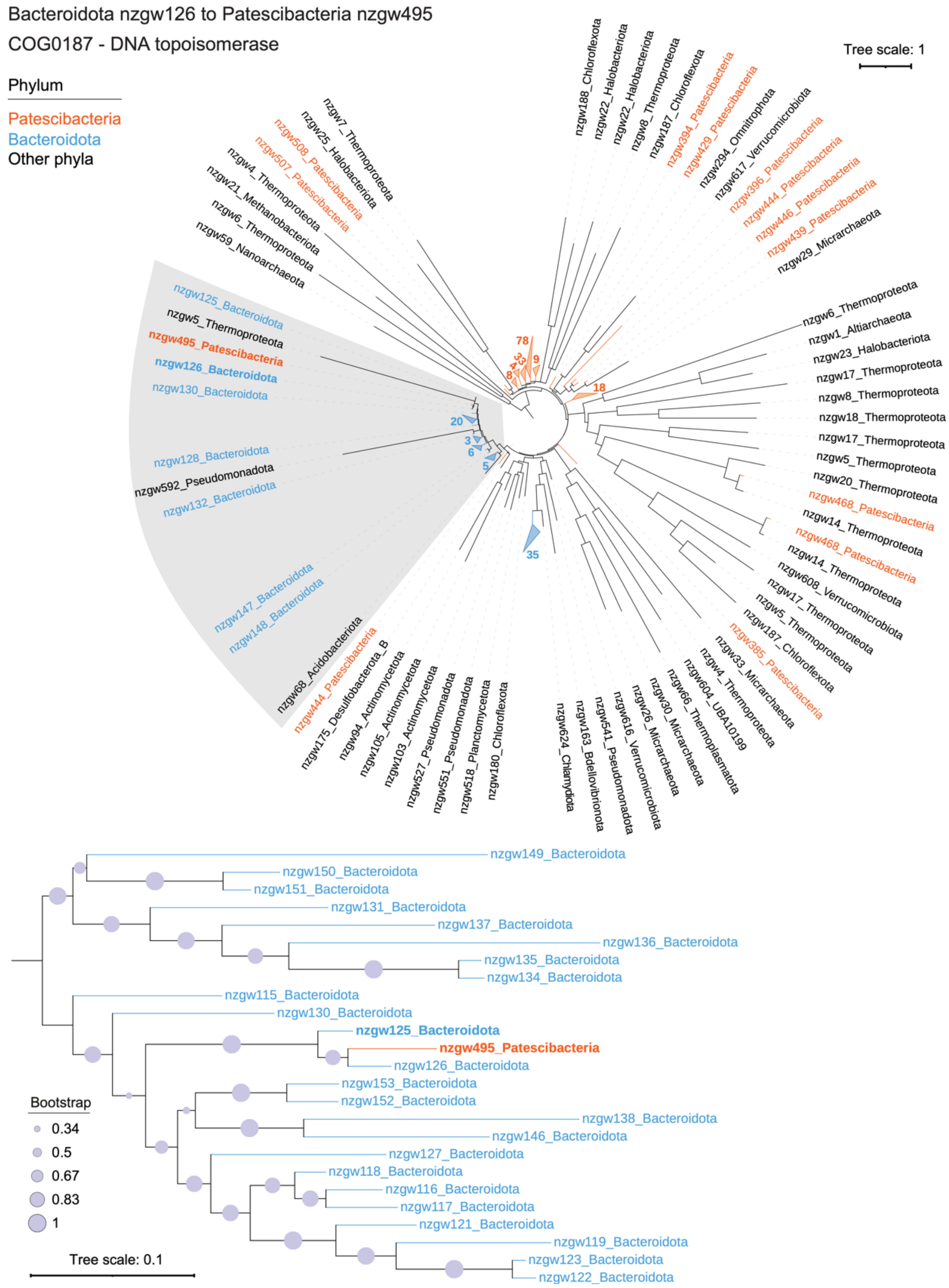

Figure S3. Phylogeny of COG0187 (DNA topoisomerase) protein sequences. Tree (top) uses all COG0187 protein sequences in this study longer than 50 amino acids long (n=893), and shows the close phylogenetic relatedness between the GyrB protein sequences encoded by Bacteroidota nzgw126 and Patescibacteria nzgw495 (in bold). Protein sequences were processed and the tree was constructed as described for the LysR tree (Fig. 5) in the methods. Clades containing three or more members of those two phyla were collapsed for better visualization, and numbers of branches within each collapsed clade are specified. The tree was rooted at the midpoint. Grey shading indicates clades that were selected for the subsetted tree (bottom). For the subsetted tree, a visual inspection of the sequence alignment was carried out and short sequences were manually removed before tree was rebuilt following protocol described above.

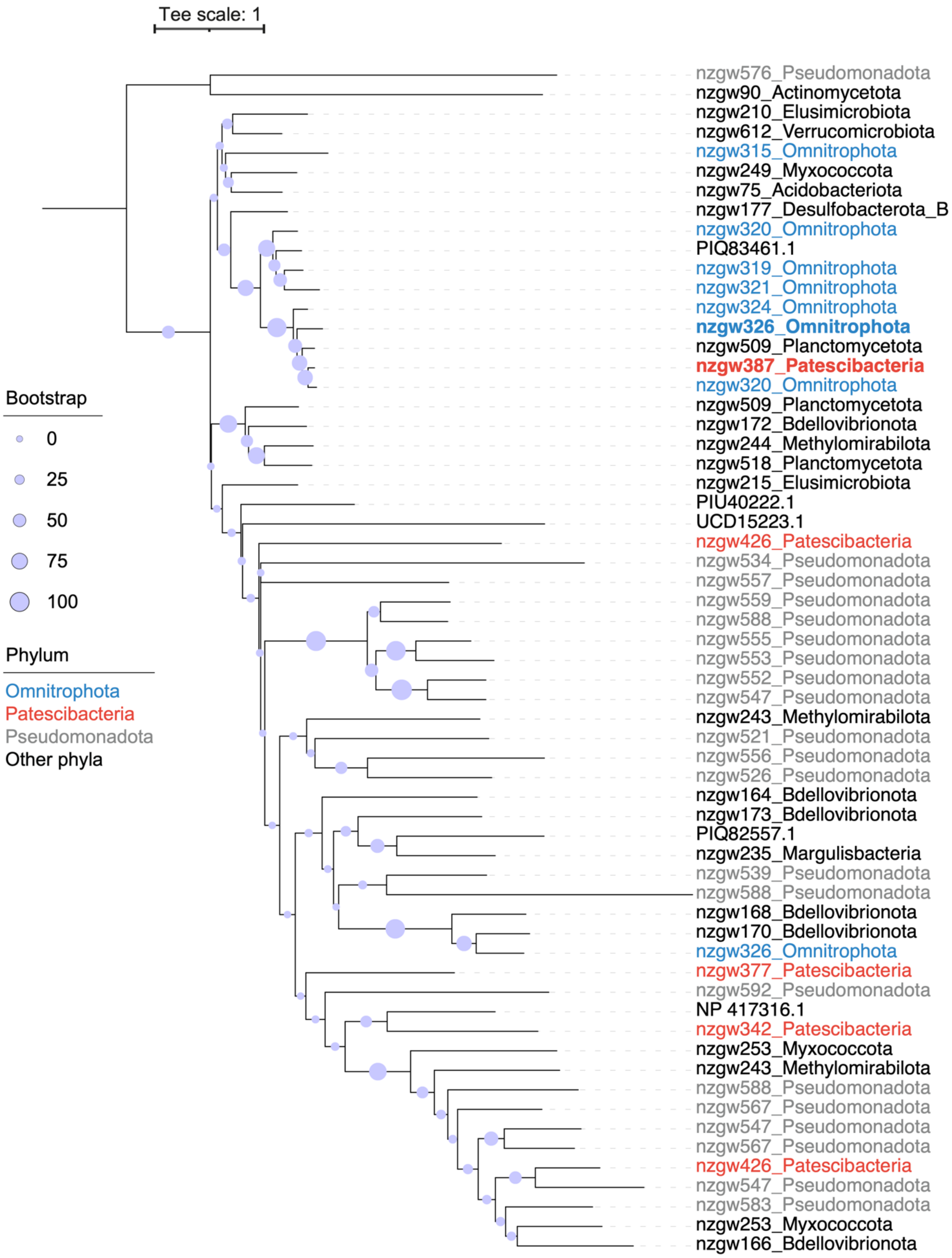

Figure S4. Phylogeny of COG0583 (LysR) protein sequences. The tree contains a subset of LysR sequences shown in figure 5, and was rebuilt using IQ-TREE. It includes all 56 LysR sequences from clusters shown in figure 5 marked by a star symbol at each cluster's base and five reference sequences as described in the methods.

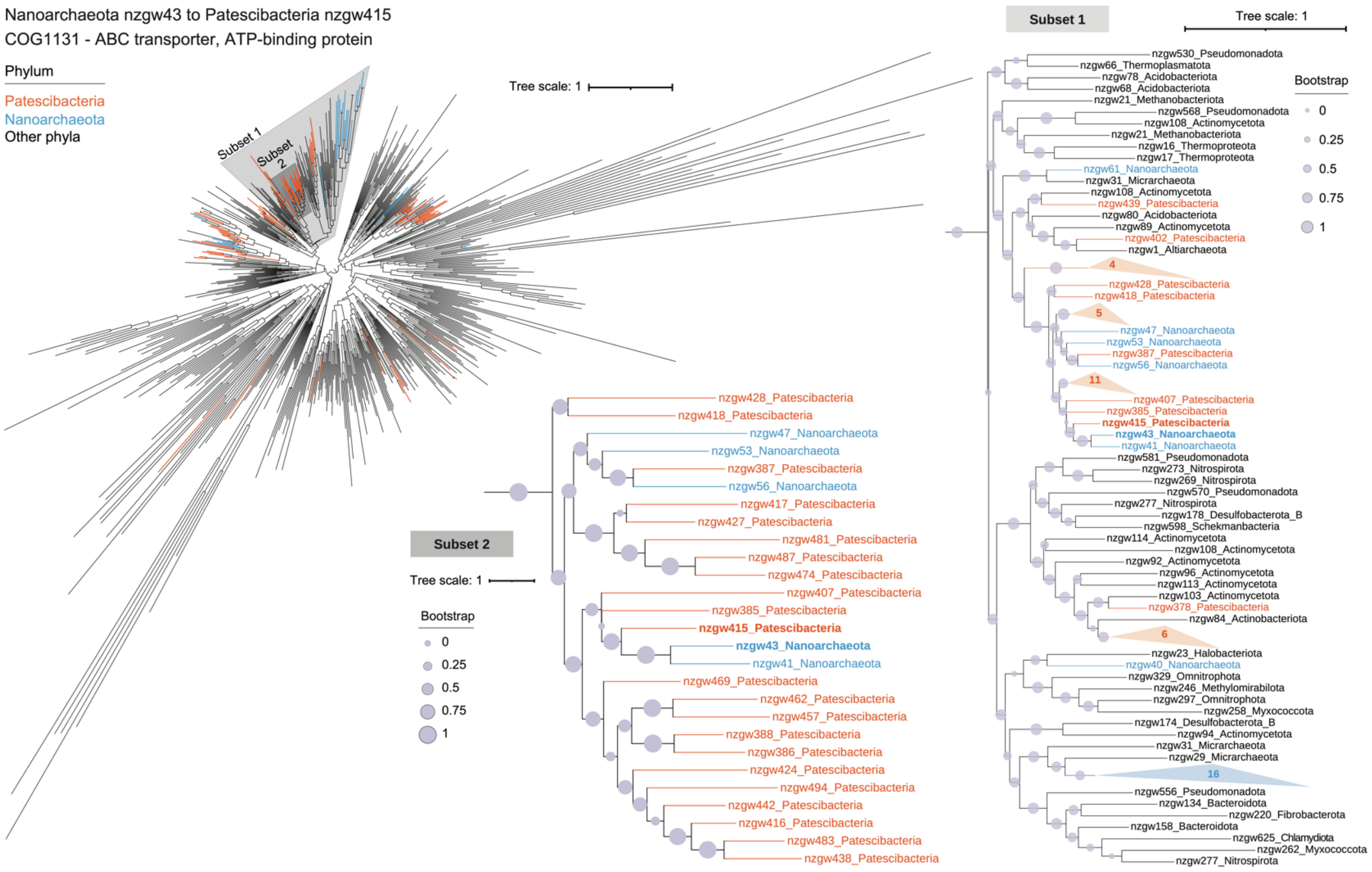

Figure S5. Phylogeny of COG1131 (ABC transporter) protein sequences. Tree (top left) uses all COG1131 protein sequences in this study longer than 50 amino acids long (n=2,336), and shows the close phylogenetic relatedness between the ABC transporter, ATP-binding proteins encoded by Nanoarchaeota nzgw43 and Patescibacteria nzgw415. Protein sequences were processed and the tree was constructed as described for the LysR tree (Fig. 5) in the methods. The tree was rooted at the midpoint. Grey shading indicates clades that were selected for subsetted trees (bottom and right). Clades containing three or more members of Patescibacteria and Nanoarchaeota were collapsed for better visualization, and numbers of branches within each collapsed clade are specified.

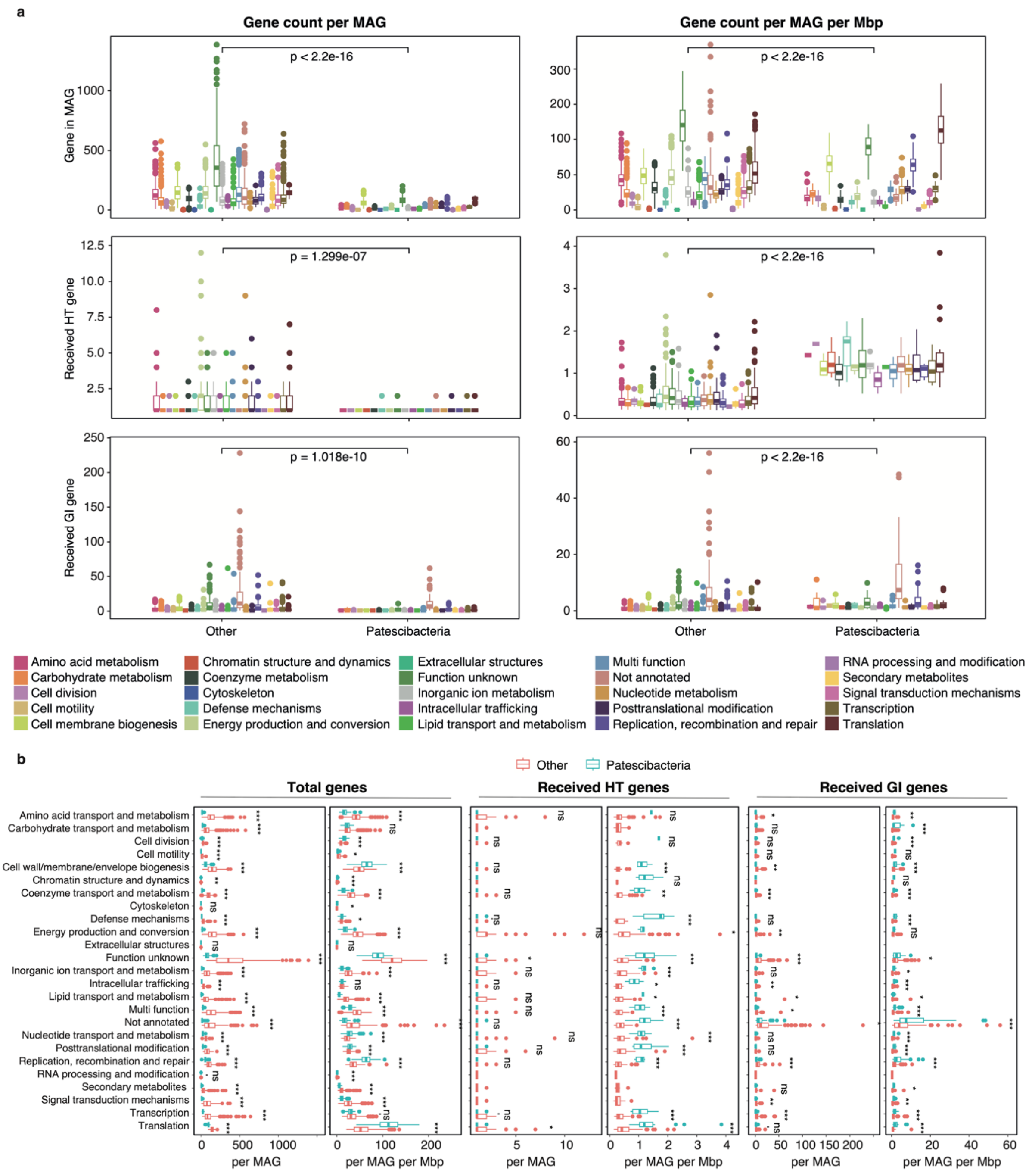

Figure S6. Difference in metabolic functions of genes received through horizontal transfer between Patescibacteria and other taxa. (a) Comparison across all metabolic categories of all genes, HT genes received and GI genes received between Patescibacteria and other taxa. (b) Comparison of each metabolic category for all genes, HT genes received and GI genes received between Patescibacteria and other taxa. For (a) and (b) each data point represents a MAG. Wilcoxon Signed Rank tests were undertaken between the overall metabolic categories of Patescibacteria and other taxa (a) or between Patescibacteria and other taxa for each metabolic category (b). P-values are ns: not significant, * <0.05), ** <0.01) and *** <0.001. Box plots show the interquartile range and median (boxes and center line), ± 1.5 interquartile range (whiskers), and data points outside these ranges.

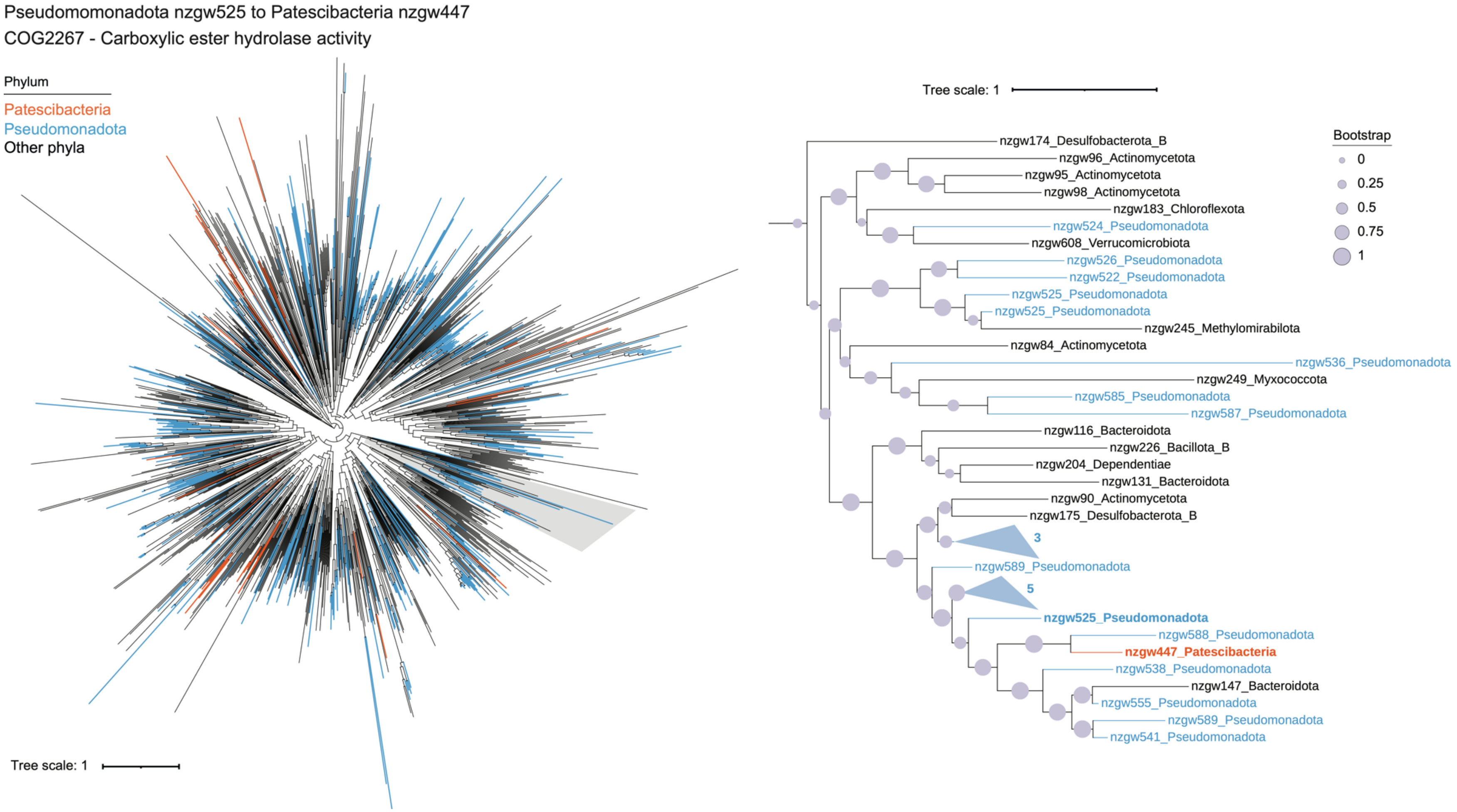

Figure S7. Phylogeny of COG2267 (lysophospholipase) protein sequences. Tree (left) uses all COG2267 protein sequences in this study longer than 50 amino acids long (n=1,928), and shows the close phylogenetic relatedness between the alpha beta hydrolase superfamily proteins encoded by Pseudomonadota nzgw525 and Patescibacteria nzgw447 (in bold). Protein sequences were processed and tree was constructed as described for the LysR tree (Fig. 5) in the methods. The tree was rooted at midpoint. Grey shading indicates clades that were selected for the subsetted tree (right). Clades containing three or more members of Pseudomonadota and Patescibacteria were collapsed for better visualization, and numbers of branches within each collapsed clade are specified.

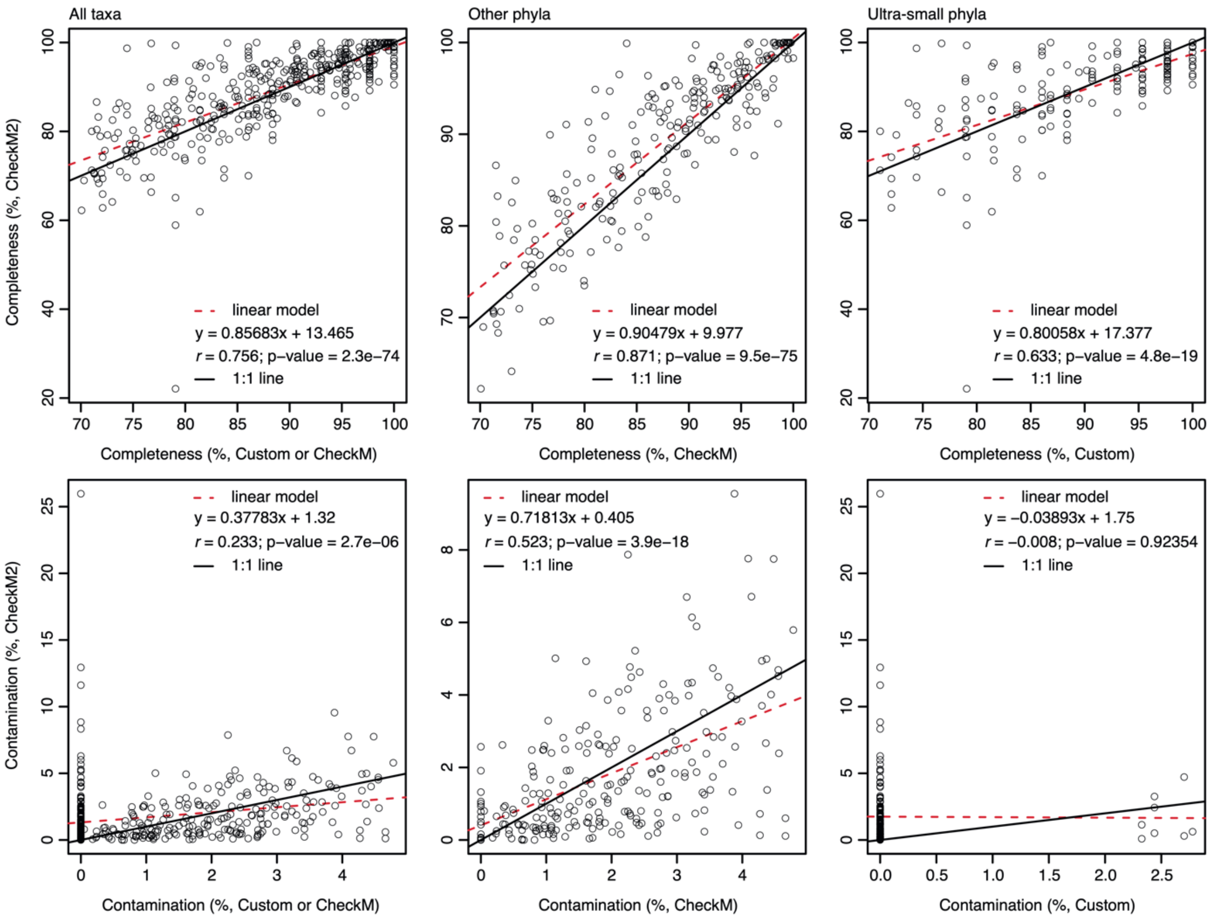

Figure S8. Comparison of MAG estimated completeness and contamination based on the SCG (custom or CheckM) or CheckM2 approach. The custom SCG approach was used for ultra-small taxa, and the CheckM SCG approach was used for other taxa. CheckM2 was applied to all taxa. Linear models are fitted to the data, and Pearson correlations are shown for each plot. Completeness estimates were strongly and significantly correlated in all cases when comparing estimates for other taxa or ultra-small taxa. For taxa overall the average estimated completeness was 87.9% ± 8.4 SD versus 88.8% ± 9.5 SD for the SCG and CheckM2 approaches, respectively. For ultra small taxa this was 88.5% ± 8.4 SD versus 88.2% ± 10.6 SD. Correlations between contamination estimates were overall poorer, but were nonetheless significant for estimates for other taxa. For ultra-small taxa there was no linear correlation, although both methods estimated <5% contamination for most MAGs (n=114/125). For taxa overall the average estimated contamination was 1.2 ± 1.4 SD versus 1.8 ± 2.2 SD for the SCG approach and CheckM2, respectively. For ultra-small taxa this was 0.1 ± 0.6 SD versus 1.7 ± 2.8 SD, respectively. Notable outliers based on comparisons with CheckM2 were MAG nzgw409 (22% estimated completeness) which did not contribute to any HGT results, and MAG nzgw400 (26% estimated contamination). MAG nzgw400 (26% estimated contamination) is predicted to have received two HT genes from two other Patescibacteria, and donated four genes (one each to a Patescibacteria, Nanoarchaeota, Omnitrophota, and Bacteroidota). Genes on either side of HT donor or recipient genes in MAG nzgw400 were checked via BLASTP searches against the NCBI NR database (up to 10 syntenous genes depending on the contig size; range 3-10 and average 7.0 ± 2.3 SD). Syntenous genes were most closely related to Patescibacteria based on the top 15 hits for all six contigs, and top hits notably included Candidatus Levybacteria for the five contigs with ≥7 syntenous genes available for inspection. Thus contigs with HT genes in MAG nzgw400 are consistent with the MAG taxonomy (Levyibacteriales) and are unlikely to represent contamination.
